# MARS2 drives metabolic switch of non-small-cell lung cancer cells via interaction with MCU

**DOI:** 10.1016/j.redox.2023.102628

**Published:** 2023-02-06

**Authors:** Juhyeon Son, Okkeun Jung, Jong Heon Kim, Kyu Sang Park, Hee-Seok Kweon, Nhung Thi Nguyen, Yu Jin Lee, Hansol Cha, Yejin Lee, Quangdon Tran, Yoona Seo, Jongsun Park, Jungwon Choi, Heesun Cheong, Sang Yeol Lee

**Affiliations:** a Department of Life Sciences, College of BioNano Technology, Gachon University, Seongnam, Gyeonggi, 13120, South Korea; b Cancer Molecular Biology Branch, Division of Cancer Biology, Research Institute, National Cancer Center, Goyang, Gyeonggi, 10408, South Korea; c Department of Cancer Biomedical Science, Graduate School of Cancer Sciences and Policy, National Cancer Center, Goyang, Gyeonggi, 10408, South Korea; d Department of Physiology, Yonsei University Wonju College of Medicine, Wonju, Gangwon, 26424, South Korea; e Electron Microscopy Research Center, Korea Basic Science Institute, Cheongju, Chungbuk, 28119, South Korea; f Department of Pharmacology and Medical Sciences, Metabolic Syndrom and Cell Signaling Laboratory, Institute for Cancer Research, College of Medicine, Chungnam National University, Daejeon, 35015, South Korea

**Keywords:** Mitochondrial methionyl-tRNA synthetase, Mitochondrial calcium uniporter, Cancer metabolism, p53, Reactive oxygen species, Epithelial-mesenchymal transition, ARS, aminoacyl-tRNA synthetase, MARS2, mitochondrial methionyl-tRNA synthetase, MCU, mitochondrial calcium uniporter, ROS, reactive oxygen species, OXPHOS, oxidative phosphorylation, PPP, pentose phosphate pathway, NSCLC, non-small-cell lung cancer, TIGAR, *TP53*-induced glycolysis and apoptosis regulator, EMT, epithelial-mesenchymal transition

## Abstract

Mitochondrial methionyl-tRNA synthetase (MARS2) canonically mediates the formation of fMet-tRNA_i_^fMet^ for mitochondrial translation initiation. Mitochondrial calcium uniporter (MCU) is a major gate of Ca^2+^ flux from cytosol into the mitochondrial matrix. We found that MARS2 interacts with MCU and stimulates mitochondrial Ca^2+^ influx. Methionine binding to MARS2 would act as a molecular switch that regulates MARS2-MCU interaction. Endogenous knockdown of *MARS2* attenuates mitochondrial Ca^2+^ influx and induces p53 upregulation through the Ca^2+^-dependent CaMKII/CREB signaling. Subsequently, metabolic rewiring from glycolysis into pentose phosphate pathway is triggered and cellular reactive oxygen species level decreases. This metabolic switch induces inhibition of epithelial-mesenchymal transition (EMT) via cellular redox regulation. Expression of MARS2 is regulated by ZEB1 transcription factor in response to Wnt signaling. Our results suggest the mechanisms of mitochondrial Ca^2+^ uptake and metabolic control of cancer that are exerted by the key factors of the mitochondrial translational machinery and Ca^2+^ homeostasis.

## Highlights

• MARS2 exerts cellular redox regulation through the p53-dependent metabolic switch in non-small-cell lung cancer cells.
• MARS2 downregulates p53 by Ca^2+^-dependent CaMKII/CREB signaling via interaction with MCU.
• EMT is stimulated by MARS2-driven cellular redox control.
• MARS2 expression is activated by ZEB1 in response to Wnt signaling.

## Graphical abstract

**Figure:**
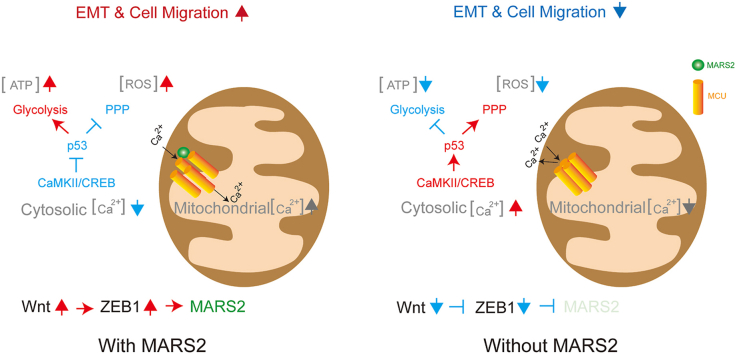

## 1 Introduction

Aminoacyl-tRNA synthetases (ARSs) are core factors of the translational machinery in both prokaryotic and eukaryotic cells. They catalyze aminoacylation reactions, wherein specific amino acids are connected to tRNAs bearing corresponding anticodon sequences. In eukaryotic cells, 9 ARSs and 3 non-enzymatic factors comprise higher order multi-tRNA synthetase complexes via multiple protein-protein interaction networks. The members of multi-tRNA synthetase complexes are involved in not only translation, but also transcription and various signaling pathways, many of which are known to be associated with human ailments such as cancer and autoimmune diseases [1,2].

Mitochondria possess their unique ARS systems for protein synthesis from their own genome. Although recessively-inherited mutations in human mitochondrial ARS have been implicated in many different human diseases, relatively little is known of the non-canonical functions of mitochondrial ARS compared to members of the ARS complex [3]. Given the significant importance of methionyl-tRNA synthetase (MARS) in translational control and DNA repair, among the mitochondrial ARSs, mitochondrial methionyl-tRNA synthetase (MARS2) is particularly interesting as it is involved in translational initiation in mitochondria by producing fMet-tRNA_i_^fMet^ [4,5].

Mitochondrial calcium homeostasis impacts a broad range of cellular events, such as metabolism, apoptosis and intracellular Ca^2+^ signaling through the calcium uptake [6,7]. Mitochondrial calcium uniporter (MCU) is located at the mitochondrial inner membrane and functions as a major gate of Ca^2+^ flux from cytosol into the mitochondrial matrix. MCU oligomerizes to form a channel for Ca^2+^ and forms a multi-protein complex that is composed of MCUR1, MCUb, EMRE, MICU1 and MICU2, which include major control units for the channel operation [8].

Recent reports have implicated mitochondria in various hallmarks of cancer including excessive proliferation, evasion of cell death, migration and dysregulation of energy metabolism [9]. Mitochondria are major generator of reactive oxygen species (ROS) as incomplete reduction of oxygen during oxidative phosphorylation (OXPHOS) induces a variety of ROS. Mitochondria can impact on cancer progression via ROS, the byproducts of energy metabolism. Excessive mitochondrial ROS production drives apoptosis and metastasis of cancer cell as mitochondrial ROS act as important signaling molecules that are related with cancer metabolism. Mitochondrial ROS may also attack nucleic acids and induce genome instability and mutations affecting tumorigenesis. These altogether highlight the ultimate importance of metabolic regulation driven by mitochondria in cancer cells.

Here, we report that MARS2 regulates mitochondrial Ca^2+^ influx via interaction with MCU. We reveal the non-canonical functions of MARS2 that control cellular redox state through the regulation of mitochondrial Ca^2+^ flux as a member of MCU complex. This report also suggests mechanisms for the mitochondrial impact on cancer progression via cellular redox regulation without controlling mitochondrial ROS generation. Knockdown of endogenous *MARS2* induces increase of cellular p53 level through the Ca^2+^-related CaMKII/CREB signaling. Subsequently, metabolic rewiring from glycolysis into pentose phosphate pathway is triggered and cellular reactive oxygen species level decreases. This metabolic switch accompanies inhibition of EMT and metastatic characteristics of cancer cells; migration, invasion, and expression of matrix metalloproteinase-2 (MMP-2). MARS2 expression is regulated by canonical Wnt signaling via ZEB1 transcription factor. These results demonstrate that mitochondria influence cancer progression through the regulation of mitochondrial Ca^2+^ flux by the core factors of the mitochondrial translational machinery and Ca^2+^ homeostasis.

## 2 Results

### 2.1 MARS2 regulates cellular redox state via p53

**Fig. 1:**
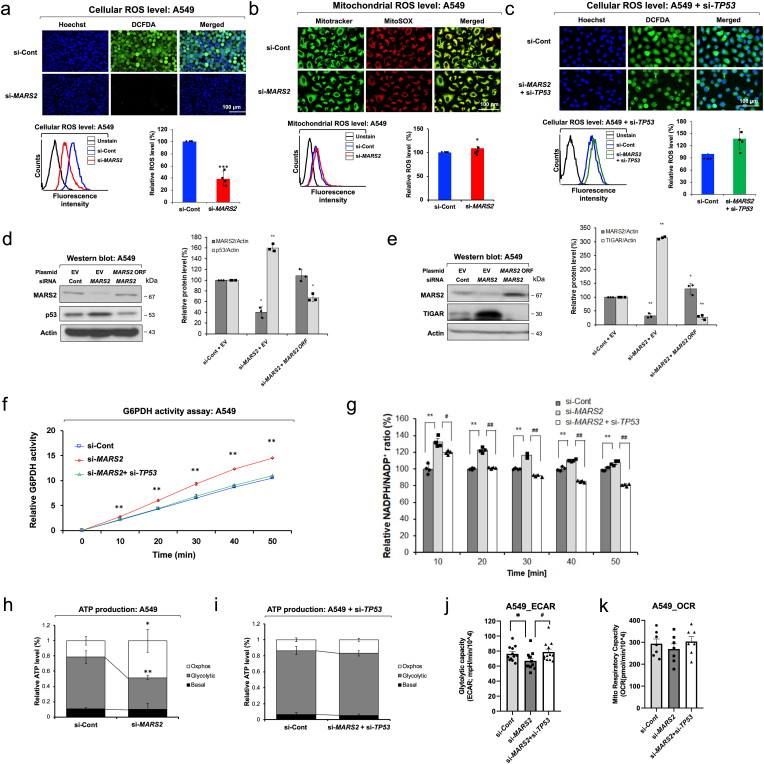
**MARS2 regulates cellular redox state via p53. a.** Cellular ROS level was analyzed by DCF-DA confocal microscopy and flow cytometry assay upon *MARS2* knockdown in A549 non-small cell lung cancer (NSCLC) cells (n = 5). si-Cont indicates si-control RNA. **b.** Mitochondrial ROS level was analyzed by MitoSox confocal microscopy and flow cytometry assay upon *MARS2* knockdown in A549 cells (n = 5). **c.** Cellular ROS level was analyzed by DCF-DA confocal microscopy and flow cytometry assay of A549 cells upon *MARS2* knockdown and *TP53* double knockdowns (n = 5). **d.** Western blot analysis of p53 protein level upon *MARS2* knockdown and a rescue assay with exogenous *MARS2* expression in A549 cells (n = 3). **e.** Western blot analysis of TIGAR protein level upon *MARS2* knockdown and a rescue assay with exogenous *MARS2* expression in A549 cells (n = 3). **f.** G6PDH activity assay of A549 cells upon *MARS*2 knockdown and double knockdowns of *MARS2* and *TP53* (n = 3). **g.** NADPH assay of A549 cells upon *MARS*2 knockdown and double knockdowns of *MARS2* and *TP53* (n = 4). **h.** ATP production profile of A549 NSCLC cells upon *MARS2* knockdown was indicated by the ratio of glycolytic ATP production level, mitochondrial ATP production level (OXPHOS) and basal level (n = 5). **i.** ATP production profile of A549 NSCLC cells upon *MARS2* and *TP53* double knockdowns was indicated by the ratio of glycolytic ATP production level, mitochondrial ATP production level (OXPHOS) and basal level (n = 5). **j.** Extracellular acidification rate (ECAR) with *MARS2* knockdown and double knockdowns of *MARS2* + *TP53* in A549 cells (n = 10). **k.** Oxygen consumption rate (OCR) with *MARS2* knockdown and *MARS2* + *TP53* double knockdowns in A549 cells (n = 7). All the quantitative data in graphs are marked as the mean ± S.D from at least three independent samples. Statistical analyses of results were performed with Student's t-test or ANOVA followed by Tukey's test (*, P < 0.05, **, P < 0.01, ***, P < 0.001, #, P < 0.05 versus si-*MARS2*, ##, P < 0.01 versus si-*MARS2*).

Modification of mitochondrial metabolism can drive the stimulation of mitochondrial ROS production and ROS-associated cancer progression. We hypothesized that cellular ROS level will decrease upon *MARS2* knockdown. Since MARS2 is involved in the translational initiation inside mitochondria, mitochondrial translation initiation is negatively affected by *MARS2* knockdown and mitochondrial ROS production would decrease. To verify our hypothesis, we investigated the change of cellular ROS level upon *MARS2* knockdown using anti-*MARS2* siRNA in A549 non-small-cell lung cancer (NSCLC) cells (Fig. 1a). As we expected, cellular ROS level decreased by approximately 50% upon *MARS2* knockdown (Fig. 1a). To check whether the decrease of cellular ROS level is the result of the suppressive effect of *MARS2* knockdown on mitochondrial ROS production, we analyzed the mitochondrial ROS level using MitoSOX (which detects mitochondrial ROS) upon *MARS2* knockdown. Contrary to our expectation, mitochondrial ROS level was not affected by *MARS2* knockdown (Fig. 1b). Mitochondrial protein synthesis, which is represented by COX2 and COX4 levels, was also not downregulated by *MARS2* knockdown in A549 cells (Fig. S1a). These results probably imply that the change of cellular ROS level induced by *MARS2* knockdown would not be related with the mitochondrial dysfunction.

When glucose-6-phosphate is formed from glucose, it may enter one of two pathways: glycolysis, where ATP and pyruvate are generated; or the pentose phosphate pathway (PPP), which is the primary source of NADPH, an important scavenger molecule of cellular ROS. p53 stimulates the expression of *TP53*-induced glycolysis and apoptosis regulator (TIGAR), which forces glucose to choose the PPP instead of glycolysis [10]. As a result, the ROS level decreases by glutathione (GSH) which is reduced from glutathione disulfide (GSSG) by glutathione reductase using NADPH from the PPP. To confirm whether p53 is a key factor for MARS2-dependent cellular redox regulation, we conducted the ROS assay with p53-suppressed A549 cells. When p53 was suppressed by anti-*TP53* siRNA, cellular ROS level were not affected by *MARS2* knockdown in A549 cells (Fig. 1c). Then, we confirmed all the results so far in H460 human lung cancer cells which also express wild type p53. When MARS2 level is suppressed by *MARS2* knockdown, cellular ROS level decreases in a p53-dependent manner without affecting mitochondrial ROS production (Fig. S1b, c & d). Mitochondrial protein synthesis is not downregulated by *MARS2* knockdown also in H460 cells (Fig. S1e). In the meantime, we observed significant increase of cellular p53 upon *MARS2* knockdown in A549 cells (Fig. 1d). This result was also observed in H460 cells (Fig. S1f). To check whether the stimulatory effect of MARS2 knockdown on p53 level is not limited to the lung cancer cells, we selected MCF7 human breast cancer cells, which also has a wild-type p53, and conducted western blot analysis of p53 upon *MARS2* knockdown. The result indicates that the stimulatory effect of MARS2 knockdown on p53 level may not be limited to the lung cancer cells (Fig. S1g). The stimulatory effect on p53 level by *MARS2* knockdown was observed regardless of the siRNA sequences (Fig. S1h). A rescue assay with exogenous MARS2 expression was also performed in western blot analysis, and the association of MARS2 with p53 expression level was verified (Fig. 1d). These results suggest the potential involvement of p53 on the MARS2-dependent ROS modulation.

We observed significant elevation of cellular TIGAR levels by *MARS2* knockdown in A549 and H460 lung cancer cells (Fig. 1e; Fig. S1i). TIGAR positively regulates glucose-6-phosphate dehydrogenase (G6PDH), which is the first rate-limiting enzyme and generator of NADPH in the PPP [10]. ROS decreases through the activation of G6PDH and the PPP by TIGAR which is activated by p53. The activity of G6PDH was stimulated by *MARS2* knockdown in a p53-dependent manner (Fig. 1f). Consistent with the result of G6PDH assay, cellular NADPH level increased by *MARS2* knockdown in a p53-dependent manner (Fig. 1g). We also checked the effect of *MARS2* knockdown on transcriptional expression of GPx1, which is an antioxidant enzyme regulated by p53. The transcriptional expression level of GPx1 also increased upon *MARS2* knockdown (Fig. S1j). These results demonstrate that MARS2 exerts cellular redox regulation via p53.

Eukaryotic cells secure their primary supply of energy (ATP) through mitochondrial OXPHOS. Cancer cells switch their major source of ATP production from OXPHOS to aerobic glycolysis; this phenomenon is called the Warburg Effect [11]. In addition, p53 regulates the metabolic program of ATP generation by slowing down glycolysis [12]. To investigate whether *MARS2* knockdown induces p53-dependent metabolic rewiring from glycolysis into PPP, we investigated the effect of *MARS2* knockdown on glycolysis. Cellular ATP production in A549 cells was predominantly dependent on glycolysis. Approximately 70% of cellular ATP production was from glycolysis (Fig. 1h). Upon *MARS2* knockdown, glycolysis was inhibited and only about 40% of cellular ATP production was from glycolysis (Fig. 1g). When p53 was suppressed by anti-*TP53* siRNA, glycolytic ATP production was not affected by *MARS2* knockdown in A549 cells (Fig. 1i). We also measured real time extra cellular acidification rate (ECAR) and oxygen consumption rate (OCR) with Seahorse XF analyzer (Fig. 1j and k). ECAR results also confirmed that glycolysis was negatively affected by *MARS2* knockdown via p53. The results were also confirmed in H460 cells (Fig. S1k, l & m). The results in this section propose that MARS2-dependent metabolic control involves p53.

### 2.2 MARS2 regulates mitochondrial Ca^2+^ influx via interaction with MCU

**Fig. 2:**
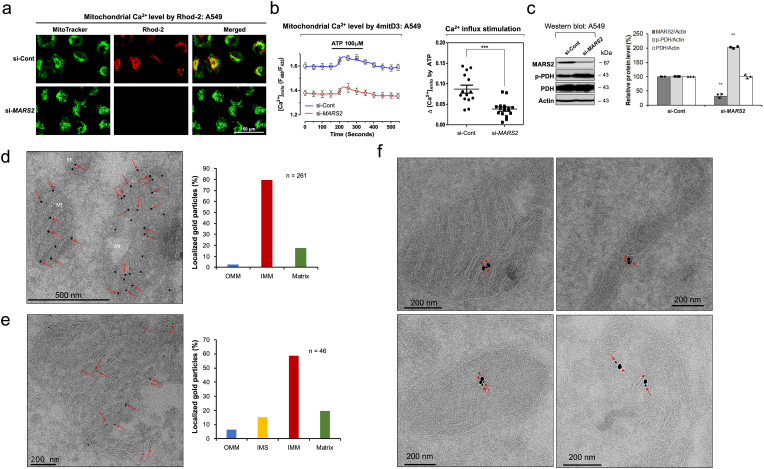
**MARS2 regulates mitochondrial Ca**^**2+**^**influx and co-localizes with MCU. a.** Mitochondrial Ca^2+^ level was visualized by confocal microscopy using Rhod-2 upon *MARS2* knockdown in A549 cells (n = 5). si-Cont indicates si-control RNA. **b.** Mitochondrial matrix Ca^2+^ level was measured using FRET-based cameleon protein probe 4mitD3, which allows ratiometric recording of emitted fluorescence from YFP (540 nm) and CFP (490 nm), in A549 cells upon *MARS2* knockdown (left) (n = 15). Stimulation of mitochondrial Ca^2+^ uptake induced by ATP (100 μM) was measured upon *MARS2* knockdown in A549 cells (right) (n = 15). **c.** Western blot analysis of PDH activation (p-PDH: inactive form of PDH), which indicates Ca^2+^ level in mitochondrial matrix, in A549 cells upon *MARS2* knockdown (n = 3). **d.** Cryo-immunogold electron microscopy of A549 cells was performed. Arrows indicate the gold particles (Diameter = 10 nm) which represent the localizations of MARS2 at inner-mitochondrial membrane (IMM) (left). Localization of each mitochondrial gold particle (n = 261) was determined and plotted its respective localization (OMM: outer mitochondrial membrane) (right). Dots indicate the gold particles which represent the localizations of MARS2. Arrows indicate the MARS2s at IMM. **e.** Cryo-immunogold electron microscopy of A549 cells was performed using smaller gold particles (Diameter = 1.4 nm). Arrows indicate the MARS2s at IMM (left). Localization of each mitochondrial gold particle (n = 46) was determined and plotted its respective localization (IMS: inter-membrane space) (right). **f.** Cryo-immunogold microscopy with double labeling using two distinct gold particles of 1.4 nm (Arrow for MARS2) and 10 nm (Arrowhead for MCU) of diameters (n = 19). All the quantitative data in graphs are marked as the mean ± S.D from at least three independent samples. Statistical analysis of results was performed with Student's t-test (***, P < 0.001).

Recently, mitochondria have caught the attention of researchers due to their ability to regulate subcellular Ca^2+^ levels [13]. In fluorescence microscopy using Rhod-2 fluorescent Ca^2+^ indicator that specifically detects the mitochondrial calcium, we observed that mitochondrial calcium level decreased by *MARS2* knockdown using anti-*MARS2* siRNA in A549 cells (Fig. 2a). The suppressive effect of *MARS2* knockdown on mitochondrial calcium level was also observed in H1299 NSCLC cells (Fig. S2a). Even though Rhod-2 is widely used as a mitochondrial Ca2+ indicator, the probe may have limitations such as accumulations in non-mitochondrial locations (e.g. cytosol or nucleus) upon staining and/or imaging conditions [14]. Therefore, to confirm the results, we measured mitochondrial matrix Ca^2+^ level ([Ca^2+^]mt) using fluorescence resonance energy transfer (FRET)-based cameleon protein probe 4mitD3, which allows ratio-metric recording of emitted fluorescence from YFP (540 nm) and CFP (490 nm), in A549 cells. As shown in Fig. 2b left, the basal level of the mitochondrial Ca^2+^ was significantly downregulated by *MARS2* knockdown. ATP-induced stimulation of mitochondrial Ca^2+^ influx also significantly decreased by *MARS2* knockdown (Fig. 2b right). The results were reconfirmed in HeLa cells, and this would indicate that the suppression of mitochondrial Ca^2+^ influx by *MARS2* knockdown is not specifically limited to A549 cells (Fig. S2b). Pyruvate dehydrogenase (PDH) in mitochondrial matrix can be inactivated when Ser293 is phosphorylated [15]. Ser293 phosphorylation of PDH is sensitive to Ca^2+^ level of mitochondrial matrix. Therefore, we also conducted western blot analysis of PDH activation upon *MARS2* knockdown (Fig. 2c). Increased p-PDH band intensity also indicates that mitochondrial Ca^2+^ level decreases when MARS2 is downregulated. These results all together suggest that MARS2 is implicated in mitochondrial Ca^2+^ influx.

MARS2 is involved in translational initiation in mitochondria by producing fMet-tRNA_i_^fMet^ [5]. To determine the submitochondrial localization of MARS2, cryo-immunogold electron microscopy of A549 cells was performed (Fig. 2d). Considering the canonical function of MARS2 as a core factor for mitochondrial translation initiation, we expected that the majority of MARS2 would localize at the mitochondrial matrix where the mitochondrial translation occurs. However, we observed that majority of MARS2 localize at mitochondrial inner membrane as represented by the distribution of the mitochondrial gold particles (Diameter = 10 nm) (Fig. 2d left). For quantitative analysis, we determined the localization of each mitochondrial gold particle (n = 261), plotted its respective localization, and came to conclude that the majority of MARS2s localizes at the vicinity of inner membrane of the mitochondria (IMM) (Fig. 2d right). To further confirm the sub-mitochondrial localization of MARS2, we employed smaller gold particles (Diameter = 1.4 nm) for the cryo-immunogold electron microscopy (Fig. 2e left). As indicated by the quantitative analysis, most of the MARS2 are found at mitochondrial inner membrane (Fig. 2e right).

**Fig. 3:**
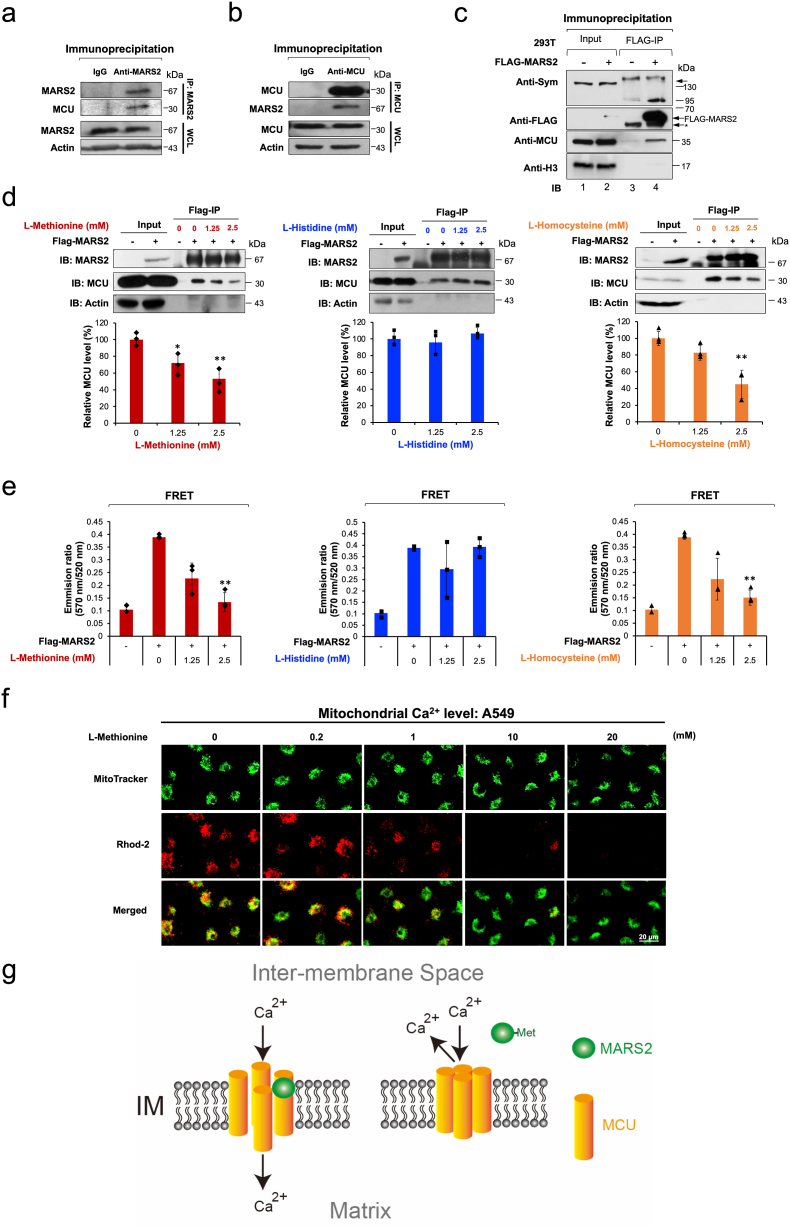
**MARS2 binds to MCU. a.** MARS2-MCU interaction was analyzed by endogenous IP assay using anti-MARS2 antibody in A549 cells (n = 3). **b.** MARS2-MCU interaction was analyzed by endogenous IP assay using anti-MCU antibody in A549 cells (n = 3). **c.** Exogenous IP assay was performed to evaluate MARS2-MCU interaction on HEK293 cells expressing FLAG-MARS2 fusion protein (n = 3). Western blot was performed with anti-MCU antibody. **d.** MARS2-MCU interaction was evaluated by exogenous IP analysis on HEK293 cells expressing FLAG-MARS2 fusion protein in the presence of increasing amount of l-Methionine, l-histidine, and l-homocysteine (0, 1.25 and 2.5 mM), respectively (left, middle and right). Western blot analyses were performed with anti-MCU antibody and the band intensities of MCU were quantitated and presented in graph (n = 3). **e.** MARS2-MCU interaction were evaluated by exogenous IP analysis on HEK293 cells expressing FLAG-MARS2 fusion protein in the presence of increasing amount of l-Methionine, l-Histidine, and l-Homocysteine (0, 1.25 and 2.5 mM), respectively (left, middle and right). FRET between MARS2-Alexa Fluor 488 and MCU-Alexa Fluor 555 was measured and presented in graph (n = 3). **f.** Mitochondrial Ca^2+^ level was visualized by confocal microscopy using Rhod-2 with the treatment of increasing dosages of l-Methionine (0, 0.2, 1, 2, 10 and 20 mM) in A549 cells (n = 6). **g.** Model for the mitochondrial Ca^2+^ influx control. Without methionine, MARS2 binds to MCU with allowing Ca^2+^ influx into mitochondrial matrix. Methionine-MARS2 binding induces dissociation of MARS2 from MCU blocks calcium influx into mitochondrial matrix. All the quantitative data in graphs are marked as the mean ± S.D from at least three independent samples. Statistical analyses of results were performed with ANOVA followed by Tukey's test. (*, P < 0.05, **, P < 0.01).

MCU is located in the mitochondrial inner membrane and functions as a major gate of Ca^2+^ flux from cytosol into the mitochondrial matrix [16]. When we tried cryo-immunogold microscopy with double labeling using two distinct gold particles of 1.4 nm (for MARS2) and 10 nm (for MCU) of diameters, the two proteins were found to co-localize at mitochondrial inner membrane (Fig. 2f). This may implicate MARS2-MCU interaction. Therefore, we tried to investigate the possible MARS2-MCU interaction with immunoprecipitation (IP) assay. From the endogenous IP assays, we were able to determine that MARS2 may interact with MCU in A549 cells (Fig. 3a and b). We also confirmed the possible interaction between MARS2 and MCU via overexpression of FLAG-tagged MARS2 and following co-IP in the HEK293T cells (Fig. 3c). The results from cryo-immunogold electron microscopy and IP assays together suggest that MARS2 interact with MCU.

The aminoacylation reaction to produce aminoacyl-tRNA is a sequential event composed of two steps. To execute the first step, MARS2 should bind methionine to catalyze the formation of methionyl-AMP. Previous reports indicated that the ARSs undergo conformational change when they bind with their specific amino acid substrates [[17], [18], [19]]. Particularly, the conformational change of *Escherichia. coli* MARS, a homologous ARS to the eukaryotic MARS2, is induced when methionine bound inside the active site pocket of MARS [18]. Based on those reports, we hypothesized that MARS2 may undergo conformational change by methionine-binding. This would affect the binding interaction of MARS2 and MCU. To test this hypothesis, the immunoprecipitated FLAG-MARS2 protein samples were treated with increasing amount of l-methionine and l-histidine (0, 1.25 and 2.5 mM) and subjected to IP assays. As seen in Fig. 3d left and middle, MARS2-MCU interaction decreased only by l-methionine treatment. This may indicate that methionine-MARS2 binding potentially controls the contact of MARS2 with MCU. In addition to methionine, l-homocysteine, another substrate for MARS2, also induces conformational change of *E. coli* MARS when it binds to active site pocket of MARS [20]. l-homocysteine treatment weakened the MARS2-MCU interaction (Fig. 3d right).

To confirm the observation that substrate binding to MARS2 weakens the MARS2-MCU binding, we treated the final bead-associated MARS2-MCU immunocomplex with secondary antibodies conjugated with Alexa Fluor 488 and Alexa Fluor 555. Then, the FRET was measured. As seen in Fig. 3e, addition of appropriate substrates (l-methionine and l-homocysteine) weakened FRET between MARS2-Alexa Fluor 488 and MCU-Alexa Fluor 555. This may reflect weaker interaction between substrate-associated MARS2 and MCU. The results together would indicate that the substrate binding to MARS2 may control the MARS2-MCU interaction. As a result, mitochondrial Ca^2+^ level decreased by the treatment of l-Methionine (0, 0.2, 1, 2, 10, 20 mM) in A549 cells (Fig. 3f). Therefore, we propose a model that the mitochondrial Ca^2+^ channel may be operated by the ON/OFF of MARS2-MCU interaction via methionine-MARS2 binding (Fig. 3g).

### 2.3 MARS2 regulates p53 via CaMKII/CREB signaling

**Fig. 4:**
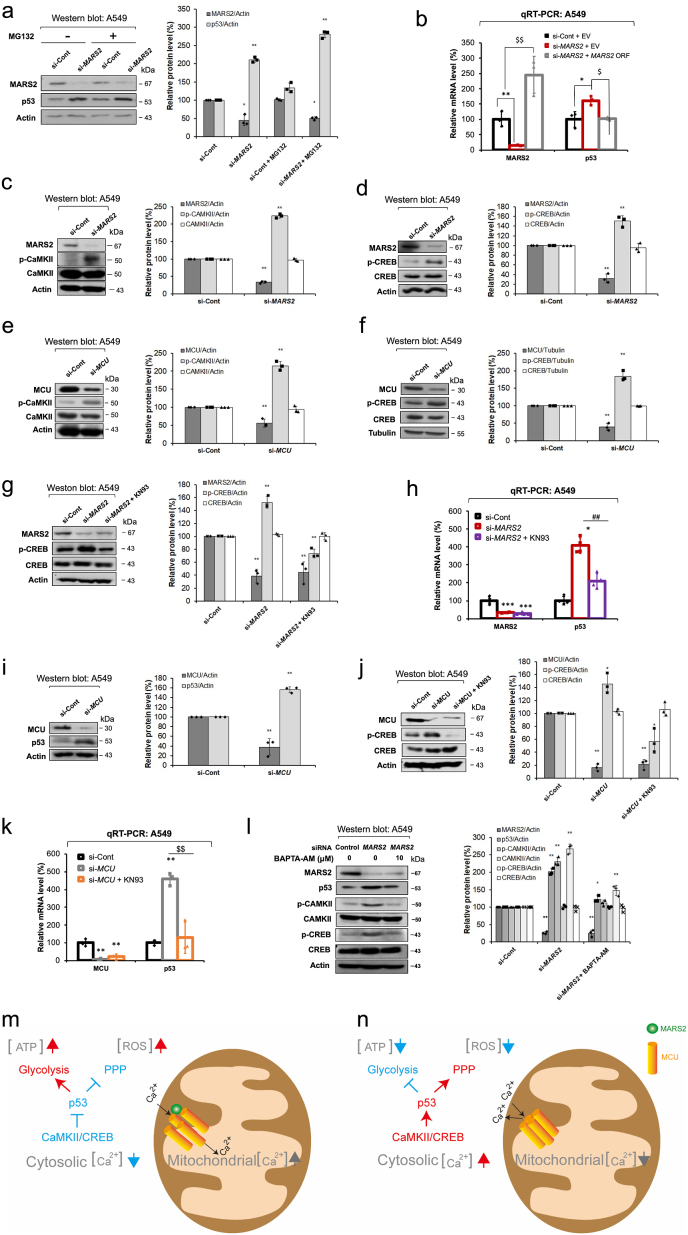
**MARS2 regulates p53 via CaMKII/CREB signaling. a.** Western blot analysis of p53 protein level in A549 cells upon *MARS2* knockdown with or without MG132 proteasome inhibitor (10 μM for 2 h) (n = 3). si-Cont indicates si-control RNA. **b.** Transcriptional expression level of p53 was evaluated by qRT-PCR with MARS2 knockdown and a rescue assay was performed with exogenous *MARS2* expression in A549 cells (n = 9). **c.** Western blot analysis of CaMKII activation (p-CaMKII: active form of CaMKII) in A549 cells upon *MARS2* knockdown (n = 3). **d.** Western blot analysis of CREB activation (p-CREB: active form of CREB) in A549 cells upon *MARS2* knockdown (n = 3). **e.** Western blot analysis of CaMKII activation in A549 cells upon *MCU* knockdown (n = 3). **f.** Western blot analysis of CREB activation in A549 cells upon *MCU* knockdown (n = 3). **g.** Western blot analysis of CREB activation of A549 cells upon *MARS2* knockdown and *MARS2* knockdown + CaMKII inhibitor KN93 (10 μM for 6 h) (n = 3). **h.** qRT-PCR analysis of transcriptional expressions of p53 in A549 cells upon *MARS2* knockdown and *MARS2* knockdown + KN93 (n = 9). **i.** Western blot analysis of p53 level in A549 cells upon *MCU* knockdown (n = 3). **j.** Western blot analysis of CREB activation of A549 cells upon *MCU* knockdown and *MCU* knockdown + CaMKII inhibitor KN93 (10 μM for 6 h) (n = 3). **k.** qRT-PCR analysis of transcriptional expressions of p53 in A549 cells upon *MCU* knockdown and *MARS2* knockdown + KN93 (n = 9). **l.** Effect of cytosolic Ca^2+^ downregulation using Ca^2+^ chelator BAPTA-AM (10 μM for 4 h) on CaMKII/CREB activation and p53 level was investigated by western blot analysis (n = 3). **m.** Model for the metabolic switch induced via CaMKII/CREB/p53 cascade by mitochondrial Ca^2+^ control of MARS2-MCU interaction. When MARS2 binds to MCU, MCU Ca^2+^ channel is activated to allow Ca^2+^ flux from cytosol into mitochondrial matrix. As a result, mitochondrial Ca^2+^ level increases and cytosolic Ca^2+^ decreases. Subsequently, p53 level decreases via CaMKII/CREB inactivation. Finally, metabolic switch from PPP into glycolysis inhibits PPP leading to ROS upregulation and promotes glycolytic ATP production. **n.** Without MARS2, MCU Ca^2+^ channel is inactivated to block Ca^2+^ flux from cytosol into mitochondrial matrix. As a result, mitochondrial Ca^2+^ level decreases and cytosolic Ca^2+^ increases. Subsequently, p53 level increases via CaMKII/CREB activation. Finally, metabolic switch from glycolysis into PPP inhibits glycolytic ATP production and promotes PPP leading to ROS downregulation. All the quantitative data in graphs are marked as the mean ± S.D from at least three independent samples. Statistical analyses of results were performed with ANOVA followed by Tukey's test. (*, P < 0.05, **, P < 0.01, ***, P < 0.001, ##, P < 0.01 versus si-*MARS2*, $$, P < 0.01 versus si-*MCU*).

Stimulation of p53 levels by *MARS2* knockdown was not affected by treatment with the 26S proteasome inhibitor MG132 (Fig. 4a). This result would exclude the possibility of proteasomal control of p53 by MARS2. Instead, MARS2 regulates p53 levels at the level of transcriptional expression as shown in the result of quantitative real-time polymerase chain reaction (qRT-PCR) in A549 lung cancer and MCF7 breast cancer cells (Fig. 4b; Fig. S3a).

Ca^2+^/calmodulin (CaM)-dependent protein kinase II (CaMKII) is a multifunctional serine/threonine kinase that is regulated by Ca^2+^ signaling [21,22]. Upon binding Ca^2+^/calmodulin complex, autophosphorylation on Thr287 induces persistent activity regardless of Ca^2+^ level and CaM association [21]. It was reported that CaMKII phosphorylates and activates several signaling molecules and transcription factors including cAMP response element-binding protein (CREB) [23,24]. In this investigation, we observed that CaMKII activation was stimulated by *MARS2* knockdown in both A549 and H1299 NSCLC cells as represented by increased p-CaMKII bands in western blot analyses (Fig. 4c & Fig. S3b). The stimulatory effect on CaMKII activation by *MARS2* knockdown was reconfirmed regardless of the siRNA sequences in A549 cells (Fig. S3c). Earlier reports indicated that the transcriptional regulation of p53 involves CREB transcription factor that is activated by CaMKII in response to Ca^2+^ [25]. Upon *MARS2* knockdown, CREB was activated in both A549 and H1299 cells (Fig. 4d & Fig. S3d). We attempted to reconfirm the MARS2-MCU interaction by examining whether the stimulations of signaling molecules by *MARS2* knockdown are mutually induced by *MCU* knockdown. Activations of CaMKII and CREB were stimulated by *MCU* knockdown as expected (Fig. 4e and f). This would demonstrate that stimulation of CaMKII/CREB signaling depends on MARS2-MCU interaction.

To clarify whether increased activity of CREB by MARS2 is exerted via CaMKII, we investigated the effect of *MARS2* knockdown on CREB activation with the treatment of CaMKII inhibitor KN93. CREB activation by *MARS2* knockdown no longer persisted when treated with KN93 (Fig. 4g). This result shows that CREB regulation by MARS2 is exerted via CaMKII. Subsequently, the stimulation of p53 transcription by *MARS2* knockdown also did not persist upon treatment with KN93, demonstrating that MARS2 controls p53 transcription via CaMKII/CREB signaling (Fig. 4h).

As seen in Fig. 4i, protein level of p53 increased also by *MCU* knockdown. The stimulations of CREB activation and p53 transcription by *MCU* knockdown were dissipated by the treatment of KN93 (Fig. 4j and k). This also demonstrates that p53 regulation by MCU is achieved through CaMKII. These together implicate that the effect of MARS2 on p53 is exerted by MARS2-MCU interaction via Ca^2+^-dependent CaMKII/CREB signaling.

The stimulatory effects of MARS2 knockdown on CaMKII, CREB and p53 were all dissipated by the treatment of BAPTA-AM, a Ca^2+^-chelating agent in A549 cells (Fig. 4l). This result would provide the evidence that CaMKII/CREB/p53 cascade is activated by the increased cytosolic Ca^2+^ level while mitochondrial Ca^2+^ level decreases upon *MARS2* knockdown. Therefore, we would propose a model that MARS2 acts as a major regulator of Ca^2+^ homeostasis between cytosol and mitochondrial matrix. This regulation would induce the metabolic rewiring between glycolysis and PPP in cancer cells (Fig. 4m and n).

### 2.4 MARS2 regulates EMT via ROS regulation

**Fig. 5:**
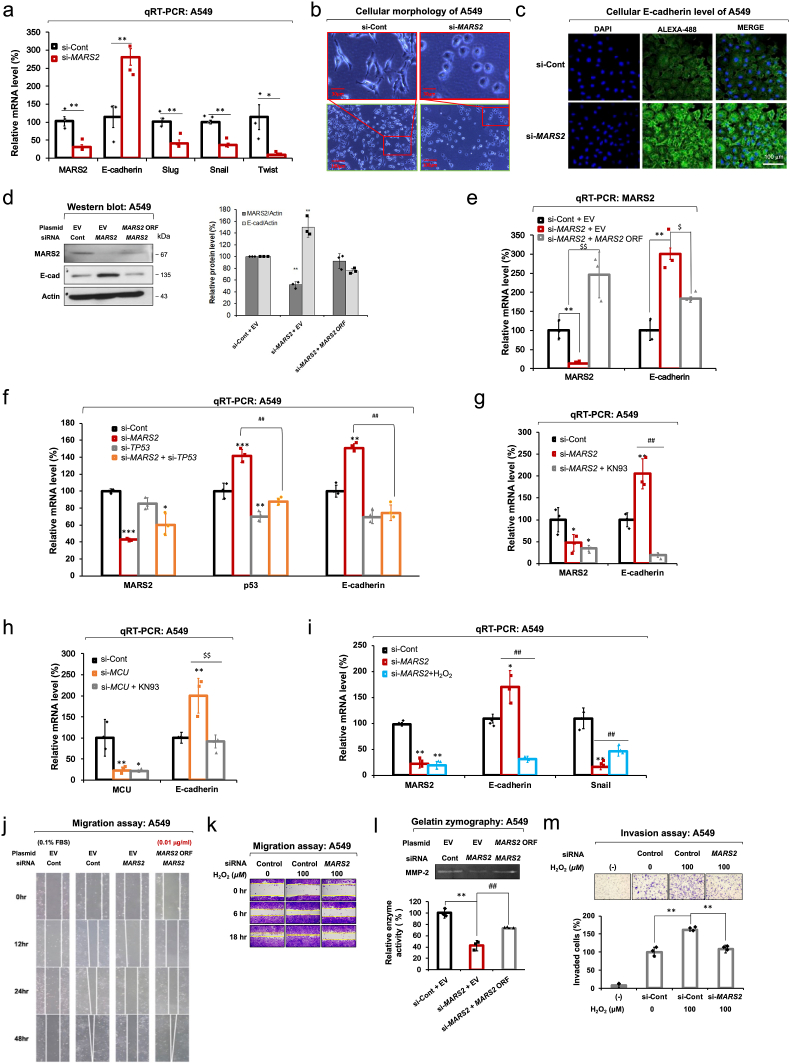
**MARS2 regulates EMT via redox regulation. a.** Transcriptional expressions of EMT markers (E-cadherin, Slug, Snail and Twist) were evaluated by qRT-PCR upon *MARS2* knockdown in A549 cells. si-Cont indicates si-control RNA (n = 9). **b.** Morphological change of TGF-β-induced A549 cells upon *MARS2* knockdown (n = 3). **c.** Confocal microscopic image of E-cadherin level in A549 cells upon *MARS2* knockdown (n = 3). **d.** Cellular E-cadherin level was checked by western blot analysis upon *MARS2* knockdown, and a rescue assay was performed with exogenous *MARS2* expression in A549 cells (n = 3). **e.** Transcriptional expression of E-cadherin was evaluated by qRT-PCR upon *MARS2* knockdown, and a rescue assay was performed with exogenous *MARS2* expression in A549 cells (n = 9). **f.** qRT-PCR of transcriptional expressions of p53 and E-cadherin in A549 cells upon *MARS2* knockdown, *TP53* knockdown and double knockdowns of *MARS2* and *TP53* (n = 9). **g.** qRT-PCR analysis of transcriptional expressions of E-cadherin in A549 cells upon *MARS2* knockdown and *MARS2* knockdown + KN93 (n = 9). **h.** qRT-PCR analysis of transcriptional expressions of E-cadherin in A549 cells upon *MCU* knockdown and *MCU* knockdown + KN93 (n = 9). **i.** Transcriptional expressions of MARS2, E-cadherin and Snail were evaluated by qRT-PCR with *MARS2* knockdown and *MARS2* knockdown + H_2_O_2_ (100 μM for 24 h) in A549 cells (n = 9). **j.** Effect of *MARS2* knockdown on A549 cell migration was investigated by wound healing cell migration assay in A549 cells (n = 3). **k.** Wound-healing cell migration assay was performed with *MARS2* knockdown in H_2_O_2_-treated A549 cells (n = 3). **l.** Activity of MMP-2 is related with MARS2 as evidenced by gelatin-zymography assay (n = 3). **m.** Invasive ability of H_2_O_2_-treated A549 cells was tested using Boyden chamber assay with *MARS2* knockdown (n = 3). Media containing 0.1% FBS (−) were used as negative controls. All the quantitative data in graphs are marked as the mean ± S.D from at least three independent samples. Statistical analyses of results were performed with Student's t-test or ANOVA followed by Tukey's test. (*, P < 0.05, **, P < 0.01, ***, P < 0.001, ##, P < 0.01 versus si-MARS2, $$, P < 0.01 versus si-*MCU*).

Ca^2+^-mediated signaling has been reported as being involved in EMT regulation in several mitochondrial retrograde signals in mammals [26]. EMT is a critical step of cancer metastasis for cancer cells to assume cellular mobility. EMT involves various cellular changes such as the weakening of cell-cell adhesion, alteration of cell-extracellular matrix (ECM) interactions, loss of cell polarity and cytoskeletal rearrangement to achieve a favorable environment for motility and invasiveness [27]. E-cadherin stabilizes cell-cell junctions and is regarded as the representative EMT-specific protein. To investigate the effect of MARS2 on EMT, we analyzed mRNA levels of key EMT markers, E-cadherin (an epithelial marker) and Slug, Snail and Twist (mesenchymal markers). As seen in Fig. 5a, mRNA level of E-cadherin significantly increased while transcriptional levels of Slug, Snail and Twist decreased upon *MARS2* knockdown in A549 cells. This result strongly implicates that the inhibition of EMT is induced by *MARS2* knockdown. Accordingly, the morphology of A549 cells was changed by *MARS2* knockdown; from the elongated spindle shape of TGF-β-induced mesenchymal cells into the rounder shape of epithelial cells (Fig. 5b). As indicated in confocal microscopy, cellular protein level of E-cadherin increases upon *MARS2* knockdown (Fig. 5c). The stimulatory effect on E-cadherin level by *MARS2* knockdown was observed regardless of the siRNA sequences (Fig. S4a). A rescue assay with exogenous *MARS2* expression demonstrated that stimulation of E-cadherin expression is a result from *MARS2* knockdown (Fig. 5d). The result from qRT-PCR indicates that MARS2 regulates E-cadherin at the transcriptional level (Fig. 5e).

In the earlier study, p53 was reported as being implicated in the transcriptional expression of E-cadherin [28]. In this investigation, the stimulation of E-cadherin expression induced by *MARS2* knockdown was also p53-dependent (Fig. 5f). Also, the stimulation of E-cadherin transcription by *MARS2* knockdown did not persist when cells were treated with KN93 (Fig. 5g). This would demonstrate that MARS2 controls E-cadherin transcription via CaMKII. Transcriptional expression of E-cadherin was also stimulated by *MCU* knockdown via CaMKII dependent manner (Fig. 5h). These together reconfirm that the effect of MARS2 on E-cadherin expression is achieved via p53 regulation that is based on the MARS2-MCU interaction.

ROS suppresses E-cadherin expression through the hypermethylation of the E-cadherin promoter by Snail up-regulation [29]. To explore whether MARS2-driven ROS regulation affects EMT, we investigated the effect of H_2_O_2_ on the expression levels of E-cadherin and Snail in *MARS2*-suppressed A549 cells. *MARS2*-dependent transcriptional increase of E-cadherin and decrease of Snail levels in A549 cells were dissipated with the treatment with H_2_O_2_, and this implicates that MARS2 regulates E-cadherin and Snail via cellular redox regulation (Fig. 5i). The results presented here so far provide the evidence that EMT is regulated by the cellular redox control of MARS2 via p53 in A549 cells.

Under the assumption that MARS2 is associated with EMT, we hypothesized that cancer cell migration and invasion would be suppressed when the expression of MARS2 is downregulated. To test our hypothesis, we tried to investigate the effect of *MARS2* knockdown on the representative characteristics of EMT in A549 cells. We observed that migration of A549 cells was inhibited by *MARS2* knockdown and a rescue assay with exogenous *MARS2* expression (Fig. 5j). Inhibition of cell migration in A549 cells by MARS2 knockdown was not persisted when the cells were treated with H_2_O_2_ (Fig. 5k). Since ECM anchors cancer cells to their original site, degradation of ECM should be preceded for cancer cells to migrate out [30]. Matrix metalloproteinase (MMP) can degrade multiple ECM protein components such as gelatin, collagen, laminin, and elastin. MMP-2 is a major gelatinase which can enzymatically degrade gelatin, and is involved in invasion of lung cancer cells [31]. It is particularly important for cancer cell migration since it not only enzymatically liberates the cancer cells from ECM by degrading gelatin but also non-enzymatically promotes the cancer cell migration via their hemopexin domain [32]. MMP-2 is also regarded as a marker molecule for EMT. In our study, MMP-2 activity in A549 cells was suppressed by *MARS2* knockdown (Fig. 5l). The transcriptional expression of MMP-2 was suppressed by *MARS2* knockdown as evidenced in the results of the qRT-PCR (Fig. S4b). The invasive ability of A549 cells was also impeded via redox regulation as evidenced by H_2_O_2_ treatment (Fig. 5m). Effects of MARS2 on cell migration and invasion via redox regulation were also confirmed in H460 cells (Figs. S4c and d).

### 2.5 MARS2 is regulated by ZEB1 in response to Wnt signaling

**Fig. 6:**
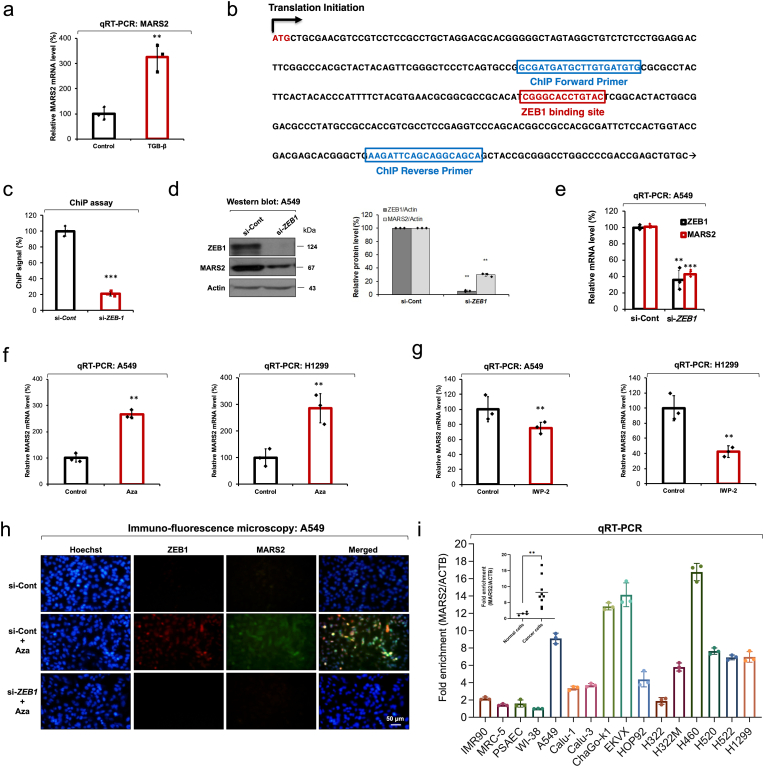
**MARS2 is regulated by ZEB1 in response to Wnt signaling. a.** Transcriptional expression of MARS2 was checked by qRT-PCR after TGF-β treatment in A549 cells (n = 9). **b.** Design of ChIP primers flanking ZEB1 binding sites near the promoter region on *MARS2* ORF. **c.** ChIP assay using ZEB1 ChIP primers on *MARS2* gene (n = 6). si-Cont indicates si-control RNA. **d.** ZEB1 regulation on MARS2 was investigated using anti-*ZEB1* siRNA by western blot analysis in A549 cells (n = 3). **e.** Transcriptional expressions of MARS2 and ZEB1 were analyzed by qRT-PCR upon *ZEB1* knockdown using anti-*ZEB1* siRNA in A549 cells (n = 9). **f.** Transcriptional expressions of MARS2 were analyzed by qRT-PCR with canonical Wnt activator 1-Azakenpaullone (Aza) treatment (10 μM for 24 h) in A549 and H1299 cells, respectively (n = 9). **g.** Transcriptional expressions of MARS2 were analyzed by qRT-PCR with canonical Wnt inhibitor IWP-2 treatment (30 μM for 24 h) in A549 and H1299 cells, respectively (n = 9). **h.** Wnt regulation on MARS2 expression was investigated with Aza and anti-*ZEB1* siRNA treatments in A549 cells by immune-fluorescence microscopy (n = 3). **i.** Comparison of MARS2 expression levels in 4 normal lung cells (IMR90, MRC-5, primary small airway epithelial cells, and WI-38) and 12 lung cancer cell lines (A549, Calu-1, Calu-3, ChaGo-k1, EKVX, HOP92, H322, H322M, H460, H520, H522, H1299) (n = 3). All the quantitative data in graphs are marked as the mean ± S. D from at least three independent samples. Statistical analyses of results were performed with Student's t-test. (**, P < 0.01, ***, P < 0.001).

When we treated A549 cells with an EMT inducer TGF-β, transcriptional expression of *MARS2* was significantly increased (Fig. 6a). Earlier report indicated that TGF-β is deeply related with *ZEB1* and *ZEB1* mRNA level increases by TGF-β treatment [33,34]. Also, a previous report indicates that ZEB1 induces EMT in lung cancer [35]. In the investigation of promoter region of *MARS2* gene, we found the ZEB1-binding site (Fig. 6b). To investigate whether ZEB1 regulates transcriptional expression of *MARS2* gene, we performed a ChIP assay using ChIP primers flanking the ZEB1-binding site on the *MARS2* gene (Fig. 6b). The ChIP assay result indicated that ZEB1 binds to the open reading frame region of *MARS2* gene (Fig. 6c). Actually, we observed that ZEB1 regulates MARS2 expression level in the western blot analysis performed using anti-*ZEB1* siRNA (Fig. 6d). mRNA level of MARS2 was significantly suppressed upon *ZEB1* knockdown using anti-*ZEB1* siRNA and this result indicates that the regulation is exerted at the transcriptional level (Fig. 6e). These results together would support that *MARS2* gene expression is regulated by ZEB1 transcription factor.

ZEB1 has been known to be a representative effecter protein of Wnt signaling [36]. In addition, it was reported that the canonical Wnt signaling regulates EMT in breast and colon, and lung cancers [[37], [38], [39]]. We investigated the effect of Wnt on the transcriptional expression level of *MARS2* to elucidate the relationship between Wnt and MARS2. We selected GSK3-β inhibitor 1-Azakenpaullone (Aza) for Wnt activation and performed qRT-PCR in A549 and H1299 cells (Fig. 6f). The transcription levels of *MARS2* were significantly increased by the treatment of Aza in both A549 and H1299 cells. On the other hand, the transcriptional expression levels of *MARS2* decreased by the treatment with the Wnt inhibitor IWP-2 in A549 and H1299 cells (Fig. 6g). As indicated in the immuno-fluorescence microscopy, ZEB1 and MARS2 levels increased by the Aza treatment (Fig. 6h). When we treated the Aza-induced A549 cells with anti-*ZEB1* siRNA, not only the ZEB1 expression level but the MARS2 expression level went down to the initial level (Fig. 6h). This result indicates that the stimulatory effect of Aza on MARS2 expression is exerted via ZEB1. The results presented in this section together propose that MARS2 expression is controlled by ZEB1 in response to canonical Wnt signaling.

Out of various tissue-specific human cancers, lung cancer is regarded as one of the most frequent and malignant one as lung cancer marks a leading cause of human death around the world. By far, two major types of lung cancers have been reported: small-cell lung cancer (SCLC) and NSCLC [40,41]. More than 80% of lung cancers are NSCLC which includes various subtypes such as large cell carcinoma, squamous carcinoma, and adenocarcinoma. To assess the possible correlation of MARS2 and cancer, we compared the MARS2 expression levels of 4 normal lung cells (IMR90, MRC-5, primary small airway epithelial cells, and WI-38) and 12 lung cancer cell lines (A549, Calu-1, Calu-3, ChaGo-k1, EKVX, HOP92, H322, H322M, H460, H520, H522, H1299) (Fig. 6i). Overexpression of MARS2 in lung cancer cells would indicate that MARS2 is associated with human lung cancer. Therefore, we tried to investigate the effect of *MARS2* knockdown on other representative characteristics of cancer progression in A549 cells. Proliferation, colony formation, viability, and apoptosis of A549 cells were not affected by *MARS2* knockdown using anti-*MARS2* siRNA (Fig. S5a∼d). This would indicate that MARS2 is associated with only with cancer metastasis. We also observed overexpression of MARS2 in human pancreatic, breast and cervical cancer cells (Figs. S5e and f). Furthermore, we also performed gene expression analysis comparing MARS2 expression between cancer and normal samples across various cancers through OncoDB (Fig. S5g). The results also indicated overexpression of MARS2 in cancer cells. These results may indicate that the close association of MARS2 with cancer metastasis is not limited to the lung cancer.

## 3 Discussion

As a main ATP supplier, the importance of mitochondria for normal eukaryotic cells have been highly appreciated so far. Although mtDNA mutations have been continuously discovered and reported in cancer cells, it has been experimentally evidenced with the mtDNA-eliminated cancer cells that the fully functional mitochondria are also required for tumor growth [42]. In this regard, the importance of MARS2 for the mitochondrial protein synthesis should be greatly valued for the optimal mitochondrial function. Considering the canonical function of MARS2 as being a core factor for mitochondrial translation initiation, the observation that MARS2 is associated with mitochondrial Ca^2+^ influx arouse our interest. For the same reason, we expected that majority of MARS2 would localize in the mitochondrial matrix, where mitochondrial translation occurs. Therefore, the sub-mitochondrial localization of MARS2, enriched distribution of MARS2 in vicinity of the mitochondrial inner-membrane which was evidenced by the cryo-immunogold electron microscopy, also drove our attention. These two observations naturally brought MCU, a major calcium channel in mitochondrial inner-membrane, up as a potential interaction partner of MARS2. MARS2 actually appeared to co-localize with MCU in the cryo-immunogold electron microscopy and the MARS2-MCU interaction was confirmed by IP assay.

In addition to the pore-forming MCU, MICU1 and its paralog MICU2 contain calcium-sensing EF-hand and reciprocally regulate MCU activity in a positive and negative way, respectively [43]. MCUR1 (40 kDa) is a transmembrane protein across the mitochondrial inner membrane and proposed to be a scaffold factor for assembly and function of MCU complex [44]. EMRE is a transmembrane protein in the mitochondrial inner membrane and required for the MCU complex function through the binding interactions with MCU and MICU1. MCUb is a paralog of MCU with 50% homology, which can bind with MCU and decrease MCU activity. These multiple proteins comprise MCU complex which is operated by gatekeeping capability with highly precise calcium sensing function. In addition to these previously reported factors of MCU complex, we propose MARS2 is another control unit of the complex. In addition, our result would implicate that methionine binding of MARS2 potentially affects the contact of MARS2 and MCU. Therefore, methionine binding to MARS2 would act as a molecular switch that regulate MARS2-MCU interaction.

CaMKII has been implicated in regulation of cellular Ca^2+^ homeostasis [45,46]. Our results indicate that CaMKII is regulated by MARS2 as represented by the increased phosphorylation at T286 upon *MARS2* knockdown. *MARS2* knockdown also induced CREB activation in a CaMKII-dependent manner. As a result, transcriptional expression of p53, a key player in cellular metabolic switch between glycolysis and PPP was stimulated. These regulations were exerted by MARS2-MCU interaction. A previous report indicated that MCU knockout facilitates NFAT activation via store-operated Ca2+ entry (SOCE)-mediated cytosolic Ca^2+^ elevation [47]. This suggests that MCU-dependent mitochondrial Ca^2+^ regulation is closely related with controlling cytosolic Ca^2+^ signals. In the other report, CaMKII activation is induced by MCU knockout while baseline cytosolic Ca^2+^ level does not change in MCU knockout cells [48]. The delayed cytoplasmic Ca^2+^ clearance may be sufficient to sustain CaMKII activity. These reports commonly emphasize the critical role of MCU in cytosolic Ca^2+^ signaling regulation via mitochondrial Ca^2+^ uptake control. The MARS2-dependent control of CaMKII/CREB signaling may also be exerted via the MCU-driven cytosolic signaling regulation.

An aspect of note in our report is the presentation of a new mechanism by which mitochondria affect glycolysis. This effect is exerted by Ca^2+^ -related p53 regulation driven by key factors of the mitochondrial translation initiation and calcium homeostasis; MARS2 and MCU. Carbohydrate metabolism to produce ATP in eukaryotic cells is a series of reaction pathways that flow subsequently from cytoplasmic glycolysis to mitochondrial OXPHOS to maximize ATP production. Glycolysis can directly affect mitochondrial ATP production by controlling the supply of pyruvate. In cellular balancing of glycolysis and PPP, p53 can influence glycolysis negatively through the regulations of the expressions of factors activating or inhibiting glycolysis [12]. p53 can also promote PPP through the stimulation of TIGAR and, subsequently, G6PDH leading to the dissipation of cellular ROS [10]. However, p53 seems to directly bind to G6PDH and inhibits G6PDH activity through the interference of the G6PDH dimerization [49]. In our G6PDH activity assay, we observed the stimulation of G6PDH activity by *MARS2* knockdown through p53. This result allowed us to conclude that G6PDH is stimulated by *MARS2* knockdown in a p53-dependent manner. G6PDH inhibition by p53 through direct binding is not particularly obvious, as indicated by reports that only 10% of G6PDH bound to p53 [49,50]. As indicated by the review by Stanton, ROS level regulation by p53 through G6PDH regulation may be dependent on the balance of positive *versus* negative effects of p53 [50].

During the EMT, epithelial cells lose their integrity and undergo a phenotypic change into mesenchymal cells. By this process, cancer cell migration and invasion are stimulated. Our data here indicated that mitochondria affect EMT through the MARS2-driven cellular redox regulation via p53 as confirmed by stimulation and inhibition of transcriptional expressions of E-cadherin and Snail, respectively. Earlier report provided the evidence that p53 suppresses canonical Wnt signaling [51]. Based on our results in this report, we propose a reciprocal phenomenon that Wnt regulates p53, which is mediated by MARS2 via ZEB1.

Deadly effects of cancer mostly arise from secondary tumors which are originated from the primary tumors via cancer metastasis. Key features that comprise cancer metastasis include EMT of cancer cells. Mitochondrial ROS can exert pro-metastatic effect via promotion of the transcriptional expression of matrix metalloproteinases, the key drivers for cancer metastasis via both enzymatic and non-enzymatic ways [52]. In this report, transcriptional expression of MMP-2 in A549 cells was regulated not by mitochondrially generated ROS but by p53-dependent cellular redox control induced by MARS2.

Although our report is focused on elucidating interaction of MARS2 with MCU and the basic mechanism by which MARS2 affects glycolysis and cellular redox control leading to EMT, an increasing number of human mitochondrial ARSs have been reported to be associated with various human diseases. Many of these are associated with neurodegenerative diseases, such as mitochondrial aspartyl-tRNA synthetase (DARS2) and mitochondrial glutamyl-tRNA synthetase (EARS2) with leukoencephalopathy, mitochondrial arginyl-tRNA synthetase (RARS2) with pontocerebellar hypoplasia, mitochondrial phenylalanyl-tRNA synthetase (FARS2) with mitochondrial encephalopathy and MARS2 with autosomal recessive spastic ataxia with leukoencephalopathy (ARSAL) in humans [3]. Since aerobic glycolysis and EMT are key characteristics of cancer progression, cancer would be a potential candidate to be defined as a human disease related with mitochondrial ARS. In this respect, further studies will be required to evaluate MARS2 and MCU as anticancer targets.

In summary, we would like to add MARS2 to the list of components of the MCU complex as a positive regulator of MCU function. We also provide a new mechanism of glycolysis control that is exerted by mitochondria. This control involves the regulation of MARS2 and mitochondrial Ca^2+^ flux to induce the Ca^2+^-related regulation of p53, which then affects EMT via redox regulation.

## Author contributions

S.Y.L. conceived and designed the study. J.S., O.J., H.S.K., N.T.N., Y.J.L., H.C., Y.L., Q.T., Y.S., and J.C. contributed to the experiments. S.Y.L., H.S.K., J.H.K., H.C. and K.S.P. analyzed the experimental data. S.Y.L. wrote the manuscript.

## Declaration of competing interest

The authors declare no conflict of interest.

## Data Availability

Data will be made available on request.

## Materials and Methods

### Cell culture

A549 human lung cancer line, MCF7 human breast cancer line, HeLa human cervical cancer cell line, HPNE human normal pancreatic cell line, 4 human pancreatic cancer cell lines (CFPAC1, MIA PaCa-2, PANC-1 and HPAFII), and human embryonic kidney (HEK) 293T cells were maintained in Dulbecco's Modified Eagle's Medium (DMEM) (HyClone, South Logan, UT, USA). 3 human normal lung cell lines (IMR90, MRC-5, and WI-38), 11 human lung cancer cell lines (Calu-1, Calu-3, ChaGo-k1, EKVX, HOP92, H322, H322M, H460, H520, H522, and H1299), and 6 human pancreatic cancer cell lines (AsPC-1, BxPC3, Capan-1, Capan-2, SNU324 and SNU410) were cultured in Roswell Park Memorial Institute 1641 medium (HyClone). All culture media were supplemented with 10% fetal bovine serum and antibiotics (100 U/mL of penicillin, and 100 mg/mL of streptomycin).

### Small interference RNA (siRNA) transfection

Cells were transfected with siRNAs using Lipofectamine 2000 (Invitrogen, Carlsbad, CA) according to the manufacturer's protocol. Sequences of siRNAs used for assays are shown in Table S1. For rescue experiments, si-*MARS2* (C) which targets 3′-UTR of *MARS2* mRNA was used. We used AccuTarget™ Negative Control siRNA (Bioneer, Daejeon, Korea) as a negative control.

### Plasmids

For overexpression of wild type MARS2 and p53, cells were transfected with pCMV6-XL6-MARS2 plasmid (OriGene, Rockville, MD, USA) and pcDNA3 p53 WT (Addgene, USA), respectively. PCR-amplified-DNA fragment encoding *MARS2* ORF was inserted to EcoR1-HindIII-treated pCMV-Tag 2B plasmid, generating pCMV-Tag 2B-MARS2 plasmid to express Flag-tagged *MARS2* ORF. For co-IP assays, HEK293T cells were transfected with pCMV-Tag 2B-MARS2 and pCMV-Tag 2B (negative control). For rescue experiments, cells were co-transfected with pCMV-Tag 2B-MARS2 and si-*MARS2* (C).

### ATP production assay

ATP measurement was conducted as previously described [53]. In brief, transfected cells were seeded in 12-well plates and incubated overnight. 20 mM of 2-deoxy-glucose (2-DG) and 20 mM of 2-DG plus 1 μM of Oligomycin A were individually treated to the cells for 3 h, followed by incubation with lysis buffer (25 mM Tris, 4 mM EGTA, 1% Triton X-100, 10% glycerol, and 2 mM dithiothreitol, pH 7.8). Lysates were mixed with reaction buffer (25 mM Tris, pH 7.8, 0.5 mM D-luciferin (Santa Cruz, USA), 1.25 μg/mL firefly luciferase (Promega, USA), 5 mM MgSO_4_, 100 μM EDTA, and 1 mM dithiothreitol). ATP level was determined using Luminometer Lumat LB 9507 and EnSpire Multilabel Plate Reader (PerkinElmer, USA). Oligomycin A blocks proton channels, resulting in the inhibition of OxPhos system-dependent ATP production while 2-DG blocks glycolysis-dependent ATP production. The intracellular ATP were calculated as follows: Glycolytic ATP (ATP_gly_) = total ATP − ATP_2-DG_, OxPhos ATP under 2-DG treatment (ATP_OxPhos_) = ATP_2-DG_ − ATP_2-DG+Oligo_.

### Cellular energy metabolism analysis

The cellular oxygen consumption rate (OCR) and the extracellular acidification rate (ECAR) were measured using the Seahorse XFe96 extracellular flux analyzer (Agilent Technologies, Santa Clara, CA, USA). Cells were plated in appropriate multi-well plates and then the cartridge filled with calibrant buffer (XF calibrant, 100840-000; Agilent Technologies) was set up on the cultured cells (8,000 cell/well/96 well plate/A549, H460), (7,000 cell/well/96 well plate/H1299). All buffers were prepared with XF base medium (10252-100; Agilent Technologies) and adjusted to reflect the pH of the cell culture medium.

For real-time measurements in living cells, the Mito-stress test kit (MST:103015-100; Agilent Technologies) was used according to the manufacturer's instructions at a final concentration of 2 μM Oligomycin, 1.5 μM FCCP, and 1 μM Rotenone/Antimycin A, respectively, during measurement. The glycolysis-stress test kit (GST: 103020-100; Agilent Technologies) was used according to the manufacturer's instructions at a final concentration of 20 mM Glucose, 2 μM Oligomycin, and 75 mM 2-DG respectively, during measurement.

Mitochondrial respiratory capacity was presented by the sum of both basal and maximal OCR rates with FCCP treatment, and then deduced the baseline OCR levels in the treatment with oligomycin. Glycolytic capacity is calculated by the combined values of glycolysis rate and maximal ECAR rate with oligomycin treatment, and then deduced the basal ECAR levels such as the treatment with either 2-DG or no glucose (Divakaruni AS, Paradyse A, Ferrick DA, Murphy AN, Jastroch M. 2014. Analysis and Interpretation of Microplate-Based Oxygen Consumption and pH data. In Methods in Enzymology, Volume 547, Chapter 16, 309–354).

### Intracellular and mitochondrial ROS detection

Transfected cells were harvested by trypsinization and resuspended in HBSS buffer. For detection of intracellular ROS detection, 10 μM of 2′, 7′-dichlorodihydrofluorescein diacetate (H_2_DCFDA) (Invitrogen, Carlsbad, CA) was added. For mitochondrial ROS detection, cells were treated with 5 μM of Mitosox (Invitrogen). Cells treated with fluorescent dyes were incubated for 10 min at incubator (37 °C) in the dark and analyzed by flow cytometry (Beckman Coulter, USA). Fluorescence by intracellular ROS was also detected by using FV3000 Confocal microscope (Olympus, Shinjuku, Tokyo, Japan).

### Western blot

Cells were lysed in RIPA buffer containing phosphatase inhibitor cocktail (Gendepot, USA) and protease inhibitor cocktail (Gendepot). Protein concentrations were normalized by BCA protein assay kit (Thermo Fisher, USA). Proteins were resuspended in sodium dodecyl sulfate (SDS) sample buffer (62 mM Tris-HCl, pH 6.8, 1 mM EDTA, 10% glycerol, 5% SDS and 50 mM dithiothreitol) and separated by SDS-PAGE. The proteins on the gel were transferred to polyvinylidene fluoride membrane (Millipore, Billerica, MA). The membranes were blocked (5% non-fat skim milk in 1 × TBS-T buffer) for 1 h at RT and incubated with primary antibodies (1:1000) at 4 °C overnight. Then, the membranes were incubated with horseradish peroxidase (HRP)-conjugated secondary antibodies (1:10000) for 2 h at room temperature (RT). Enhanced chemiluminescence (ECL) method was used to detect protein levels. List of primary antibodies are shown in Table S2.

### Glucose-6-Phosphate Dehydrogenase (G6PDH) activity assay

G6PDH activity was assessed using Glucose-6-Phosphate Dehydrogenase Activity Colorimetric Assay Kit (BioVison, CA, USA) as per the manufacturer's protocol. Briefly, cells were collected by trypsinization and resuspended in G6PDH assay buffer, substrate, and developer. Samples were transferred to 96-well plates and absorbance at 450 nm was measured using EZ Read 400 (Biochrom Ltd., Cambridge, UK).

### NADPH assay

The relative NADPH/NADP^+^ ratio was measured using NADP^+^/NADPH-Glo Assay kit (Promega, WI, USA) as per the manufacturer's protocol. Cells were harvested and prepared using assay reagents from assay kit. An equal volume of NADP^+^/NADPH-Glo detection reagent and cell samples were added to 96-well plates. After 30–60 incubation at RT, luminescence was measured using an EnSpire Multimode Plate Reader (Perkin-Elmer, MA, USA).

### Fluorescence microscopy for mitochondrial calcium

Cells were seeded on 35 mm confocal dishes and transfected with siRNAs for 72 h. Next, cells were treated with working solution [10 μM dihydrorhod-2 AM (Invitrogen), 2 μM MitoTracker Green FM (Invitrogen) and 0.02% pluronic F-127 (Sigma-Aldrich) in HBSS] for 30 min at RT protected from light. Working solution was removed and incubated with fresh HBSS for 30 min at RT. Localization of mitochondria and mitochondrial calcium level were observed by using FV3000 Confocal microscope.

### Mitochondrial calcium uptake assay

To measure mitochondrial matrix Ca^2+^ level ([Ca^2+^]mt), we used FRET-based cameleon protein probe 4mitD3, which allows ratiometric recording of emitted fluorescence from YFP (540 nm) and CFP (490 nm). Briefly, cells were seeded in 6-well plate, then transfected with siRNAs. After 24 h, cells were transfected with 4mitD3 plasmid using X-tremeGENE (Roche Diagnostics GmbH, Mannheim, Germany), then transferred to 12 mm coverslips. 48 h after plasmid transfection, cells were excited at 440 nm by using the Nipkow spinning disk confocal microscopic system, and the 4mitD3 fluorescence intensity ratio derived from measuring two emission wavelengths (F_540_/F_490_), was determined using MetaFluor 6.1 software.

### Cryo-immunogold electron microscopy

Cryo-immunogold electron microscopy was performed as previously described [54]. Briefly, A549 cells were fixed with 4% PFA and 0.05% glutaraldehyde in 0.1 M phosphate buffer with pH 7.4. The fixed cells were then embedded in 10% gelatin. Small gelatin blocks containing cells were infused with 2.3 M sucrose for overnight and then frozen in liquid nitrogen. Ultrathin cryosections (60 nm) were cut at −120 °C with a cryo-ultramicrotome (UC7/FC7, Leica, Vienna, Austria). Ultrathin sections were obtained using a diamond knife with 2.3 M sucrose: 2% methylcellulose (1:1) and transferred onto Formvar-coated copper grids.

All antibodies and gold conjugates were diluted in 0.1% bovine serum albumin (BSA)-c (Aurion, the Netherlands) or a blocking solution containing 5% BSA, 50 mM NH_4_Cl, and 0.1% saponin in PBS. The mouse monoclonal anti-MARS (1:5, 1:200), and the rabbit monoclonal anti-MCU (1:20) were used as primary antibodies, respectively. The anti-mouse IgG (1:50) was used as bridging antibody. Protein A-gold (from the Department of Cell Biology, Utrecht School of Medicine, Utrecht, the Netherlands), nanogold-conjugated Fab fragments of an anti-mouse IgG and an anti-rabbit IgG (NanoProbes, Yaphank, NY) were used to detect primary antibodies.

For the immunogold labeling experiments, the ultrathin cryosections were incubated with primary antibody for 30 min after blocking with 0.1% cold fish gelatin and 5% BSA for 20 min. The sections were then incubated with anti-mouse IgG for 30 min. Then, protein A-gold was consecutively applied. After finishing the antibody labeling step, grids were then stained with 4% neutral uranyl acetate and embedded in 2% methyl cellulose containing 0.2% uranyl acetate as described by Tokuyasu [55].

Next, the cryosections were fixed with 4% PFA in 0.1 M phosphate buffer and prepared as for double immunogold labeling. After blocking with 5% BSA, 50 mM NH4Cl, and 0.1% saponin in PBS, cryosections were incubated with primary first antibody and second antibody for 2 h. Next, the cryosections were incubated for 1 h with nanogold-conjugated Fab fragments of an anti-rabbit IgG and then washed and fixed with 1% glutaraldehyde for 5 min. Gold particles were enhanced for 4 min. Subsequently, cryosections were incubated for 1h with nanogold-conjugated Fab fragments of an anti-mouse IgG and then continued all steps as before. Gold particles were enhanced for 1 min. GoldEnhance (NanoProbes) was used to enlarge gold nanoparticles according to the manufacturer's instructions. Grids were examined at 120 kV using the KBSI Bio-HVEM System (JEM-1400 Plus (JEOL, Japan)).

For negative controls, we performed the microscopy without primary antibodies.

### Immunoprecipitation (IP)

For the endogenous IP, cells were harvested with cold PBS containing protease inhibitor. Collected cells were treated with IP lysis buffer (25 mM Tris, 15 mM NaCl, 1 mM EDTA, 1% NP-40, 5% glycerol, pH 7.4), sonicated for 30s, and centrifuged. Lysates were incubated with anti-MCU or anti-MARS2 antibodies at 4 °C overnight in a seesaw shaker. Next, lysates were treated with Protein-A Agarose (Upstate Biotechnology Inc., Lake Placid, NY) for 2 h at 4 °C. After centrifugation (3,000 rpm, 4 °C), supernatants were discarded, and beads were washed with PBS. Final immunoprecipitated beads were resuspended in a pertinent amount of PBS and mixed with SDS sample buffer, followed by Western blot analysis.

For the exogenous IP, Flag-MARS2-overexpressed HEK 293T cells were harvested and crushed in IP lysis buffer [150 mM NaCl, 25 mM HEPES-KOH (pH 7.5), 10% (v/v) glycerol, 1 mM MgCl2, 2 mM sodium orthovanadate, 2 mM β-glycerophosphate, 1 mM PMSF, 1 mM dithiothreitol, 2 mM EDTA, 0.5% Triton X-100 and 1 × protease inhibitor cocktail (Roche)]. After brief homogenization and sonication, lysates were centrifuged at 16,000×*g* for 5 min to remove insoluble materials and then incubated with anti-FLAG M2 affinity gel (Sigma-Aldrich) for 2 h at 4 °C. The collected beads were washed and treated with l-Methionine, l-Homocysteine, and l-Histidine for 2 h at 4 °C. Final immunoprecipitated beads were washed by 0.05% Triton X-100, followed by Western blot and FRET assays. For FRET measurement of MARS2-MCU complex, beads were incubated with secondary antibodies conjugated with Alexa Flour 488 (Thermo Fisher) and Alexa Flour 555 (Thermo Fisher) for 2 h at RT (1:250). Final beads were carefully washed, resuspended, and transferred to black 96-well plates. Emission intensities at 520 nm (Alexa Flour 488, donor) and 570 nm (Alexa Flour 555, acceptor) were measured by 488 nm excitation using EnSpire Multilabel Plate Reader (PerkinElmer). Emission ratio (520 nm/570 nm) was calculated to analyze FRET intensity by MARS2-MCU complex.

### Quantitative real-time polymerase chain reaction (qRT-PCR)

RNA extraction was performed using RNeasy kit (QIAGEN, Hilden, Germany) or TRIzol (Invitrogen) as per the manufacturer's protocol. Total RNA was reverse transcribed by cDNA Synthesis Kit (Philekorea, Korea) or GoScript™ Reverse Transcriptase (Promega) with random hexamer primer (Thermo Scientific Scientific) according to the manufacturer's instructions. QuantiSpeed SYBR Kit (Philekorea) with Rotor-Gene Q (QIAGEN) and Luna® Universal qPCR Master Mix (New England Biolabs) with MIC4 qPCR Cycler (Bio Molecular Systems, Queensland, Australia) were used to conduct qRT-PCR assays. Cycle threshold (Ct) values were determined, and 2ΔΔCt method was used for the analysis of relative gene expression. Primer sequences used for qRT-PCR are shown in Table S3.

### Cellular Morphology

To analyze morphological change after *MARS2* knockdown, EMT of A549 cells was induced by 5 ng/ml of TGF-β (Peprotech, Rocky Hill, NJ, USA) for 48 h, followed by *MARS2*-siRNA transfection. Cell morphology of transfected cells was observed using CKX53 inverted microscope (Olympus).

### Immuno-fluorescence microscopy

Cells seed on coverslip glass were fixed with 4% paraformaldehyde. Then, cells were permeabilized with 1% Triton X-100 in PBS at 37 °C for 30 min. Cells were covered with blocking solution (5% BSA in PBS) at RT for 1 h and. Cells were incubated with primary antibodies (1:100) in blocking solution at 4 °C overnight and washed by TBS (50 mM Tris, 150 mM NaCl, pH 7.4). Cells were treated with Alexa Flour-488 and Alexa Flour-555 conjugated secondary antibody (1:250) protecting from light at RT for 1 h. Then, cells were counterstained by DAPI or Hochest 33342 (1:2000). Coverslip was sealed with a mounting solution (SlowFade antifade Reaent). Imaging of E-cadherin was monitored and photographed by a Nikon A1 laser scanning confocal microscope. We observed immunostaining of MARS2 and ZEB1 using CKX53 inverted microscope equipped with CKX3-RFA fluorescence illuminator.

### Cell viability and proliferation assays

In cell viability assay, cells were seeded in 96-well cell culture plates (5 × 10^3^ cells/well) and transfected with siRNAs for 72 h. After transfection, old media were replaced with fresh media containing 10% cell counting kit-8 (CCK-8) (Dojindo, Rockville, MD, USA) solutions. After 1 h, relative cell viability was analyzed by EZ Read 400 to measure absorbance at 450 nm. In cell proliferation assay, transfected cells were harvested and seeded in 96-well cell culture plates (5 × 10^3^ cells/well). After 0, 24 and 48 h, relative number of cells at each time point was measured using CCK-8 assay.

### Colony formation assay

Transfected cells were harvested and cultured in 6-well cell culture plates (3 × 10^3^ cells per well). Cells were incubated for one week, exchanging culture media every 3 days. Cells were fixed with 4% paraformaldehyde and stained with 0.1% crystal violet solutions to count colonies.

### Apoptosis analysis

FITC Annexin V apoptosis detection kit (BD Bio-Sciences, Franklin Lakes, NJ, USA) was used to measure apoptosis of cells as per the manufacturer's protocol. Briefly, transfected cells were harvested and washed by PBS. Then, cells were treated with Annexin V and Propidium Iodide for 15 min at RT in a dark room. Flow cytometry (Beckman Coulter, USA) was used to detect the live cells and apoptotic cells.

### Wound healing migration assay

Transfected cells were seeded in 96-well plates at 2 × 10^4^ cells per well. The middle of the cell surface was scraped with a sterile 200 μl micropipette tip to make a scratch of constant width. Then, the debris was washed with PBS and cells were cultured in DEME containing 1% FBS and 0.1% FBS was used for the negative control. Wound closure was monitored and photographed by using CKX53 inverted microscope.

### Boyden chamber invasion assay

8 μm pore polycarbonate membrane (Neuro Probe, Gaithersburg, MD, USA) was incubated with solution containing 0.1 g/l of gelatin and 0.1% acetic acid for 2 h at RT. Transfected cells were harvested by trypsin and resuspended in DMEM containing 0.1% FBS. Media with 1% FBS were added to the lower chamber as a chemo-attractants. Then, cells were seeded on the upper chamber at a density of 4.0 × 10^4^ cells/well in 50 μl of 0.1% FBS DMEM. The chamber was incubated for 20 h at 37 °C CO_2_ incubator. After carefully removing cells on the upper surface of the membrane with Kimwipes, cells were fixed with 4% paraformaldehyde and stained with 0.1% crystal violet solutions. The invasive cells on the lower surface of the membrane were monitored by using CKX53 inverted microscope. The numbers of invaded cells were calculated in random areas of the membrane.

### Zymography

To harvest secreted MMPs, transfected cells were treated with media containing 0.1% FBS for 48 h. Conditioned media were separated by SDS-PAGE using polyacrylamide gels mixed with 0.15% gelatin from porcine skin (Sigma Aldrich). After electrophoresis, gels were rinsed with 2.5% Triton-X 100 for 30 min at RT twice to remove SDS. Renatured gels were incubated with developing buffer (50 mM Tris-HCl and 5 mM CaCl_2_; pH 7.6] for 24 h at 37 °C. Gels were stained with Coomassie Blue R250 for 10 min at RT and washed by destaining solution (20% methanol and 10% glacial acetic acid) to observe white bands which indicate the gelatinase activities by MMP-2.

### Chromatin immunoprecipitation (ChIP)

Transfected A549 cells were crosslinked with 1% formaldehyde for 10 min at RT and treated with 125 mM glycine to quench formaldehyde. Sonication was used to shear genomic DNA to a size of 100–500 base pairs. Supernatants containing protein-DNA complex were incubated with 1 μg of ZEB1 primary antibody overnight at 4 °C. Next, supernatants were treated with Protein-A Agarose for 2 h at 4 °C in a seesaw shaker. DNA was eluted by elution buffer (1% SDS and 0.1M NaHCO_3_, pH 8.0) and treated with 0.3 M NaCl overnight at 65 °C to reverse the crosslinks. DNA was purified using QIAquick PCR Purification Kit (QIAGEN) according to the manufacturer's instructions. Isolated DNA was used for qRT-PCR to detect *MARS2*-encoding sequences which interact with ZEB1.

### Gene expression analysis

To analyze the aberrant expression of MARS2 in cancer, gene expression analysis was performed using OncoDB. OncoDB assisted in data integration of patient gene expression data from The Cancer Genome Atlas (TCGA) and Genotype-Tissue Expression (GTex) samples. MARS2 expression was assessed as normalized Transcripts Per Million (TPM) and the data was re-plotted as a boxplot using GraphPad Prism (version 8.0.2).

### Statistical analysis

All the quantitative data in graphs are marked as the mean ± S.D from at least three independent samples. For two groups, Student's t-test was conducted to determine statistical significances. For more than two groups, ANOVA and Tukey's test were used.
