# Supplementary material for: MARS2 drives metabolic switch of non-small-cell lung cancer cells via interaction with MCU
Source: Redox Biol. 2023 Feb 6;60:102628. doi: 10.1016/j.redox.2023.102628 (PMC9947422; doi:10.1016/j.redox.2023.102628)
Supplement: Multimedia component 1 [file mmc1.docx]

**Supplemental Information**

**
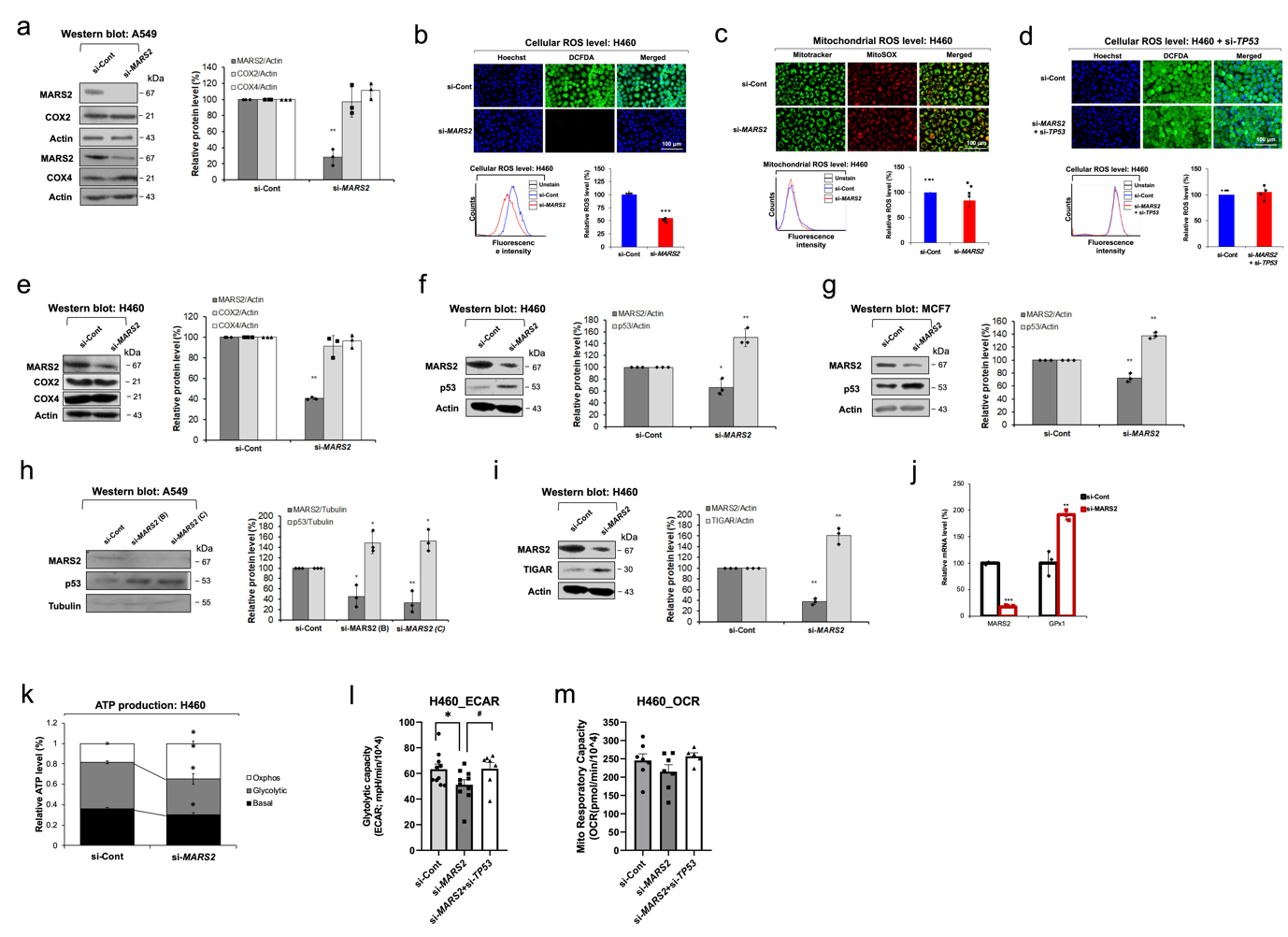
**

**Figure S1. MARS2 regulates cellular redox state via p53. a.** Mitochondrial protein synthesis was not affected by *MARS2* knockdown in A549 cells. Protein levels of COX2 and COX4, two proteins encoded in mitochondrial genome, were evaluated by western blot analysis with *MARS2* knockdown in A549 cells (n = 3). **b.** Cellular ROS level was analyzed by DCF-DA confocal microscopy and flow cytometry assay upon *MARS2* knockdown in H460 cells (n = 5). si-Cont indicates si-control RNA. **c.** Mitochondrial ROS level was analyzed by MitoSox confocal microscopy and flow cytometry assay upon *MARS2* knockdown in H460 cells (n = 3). **d.** Cellular ROS level was analyzed by DCF-DA confocal microscopy and flow cytometry assay of H460 cells upon *MARS2* knockdown and *TP53* double knockdowns (n = 3). **e.** Mitochondrial protein synthesis was not affected by *MARS2* knockdown in H460 cells. Protein levels of COX2 and COX4, two proteins encoded in mitochondrial genome, were evaluated by western blot analysis with *MARS2* knockdown in H460 cells (n = 3). **f & g.** p53 levels are up-regulated by *MARS2* knockdown in H460 human lung cancer and MCF7 human breast cancer cells. Protein levels of p53 were evaluated by western blot analysis with *MARS2* knockdown in H460 (f) and MCF7 (g) cells (n = 3). **h.** Stimulatory effect on p53 level by *MARS2* knockdown was reconfirmed with two different siRNAs. Protein level of p53 was evaluated by western blot analysis with *MARS2* knockdown using two anti-*MARS2* si-RNAs with different nucleotide sequences (n = 3). **i.** TIGAR level is up-regulated by *MARS2* knockdown in H460 cells (n = 3). **j.** Transcriptional expression level of GPx1 was evaluated by qRT-PCR with *MARS2* knockdown in A549 cells (n = 6). **k.** ATP production profile of H460 cells upon *MARS2* knockdown was indicated by the ratio of glycolytic ATP production level, mitochondrial ATP production level (OXPHOS) and basal level (n = 5). **l.** Extracellular acidification rate (ECAR) with *MARS2* knockdown and double knockdowns of *MARS2* + *TP53* in H460 cells (n = 10). **m.** Oxygen consumption rate (OCR) with *MARS2* knockdown and *MARS2* + *TP53* double knockdowns in H460 cells (n = 7). All the quantitative data in graphs are marked as the mean ± S.D from at least three independent samples. Statistical analyses of results were performed with Student’s t-test or ANOVA followed by Tukey’s test. (*, P< 0.05, **, P< 0.01, ***, P< 0.001, #, P<0.05 versus si-*MARS2*).

**
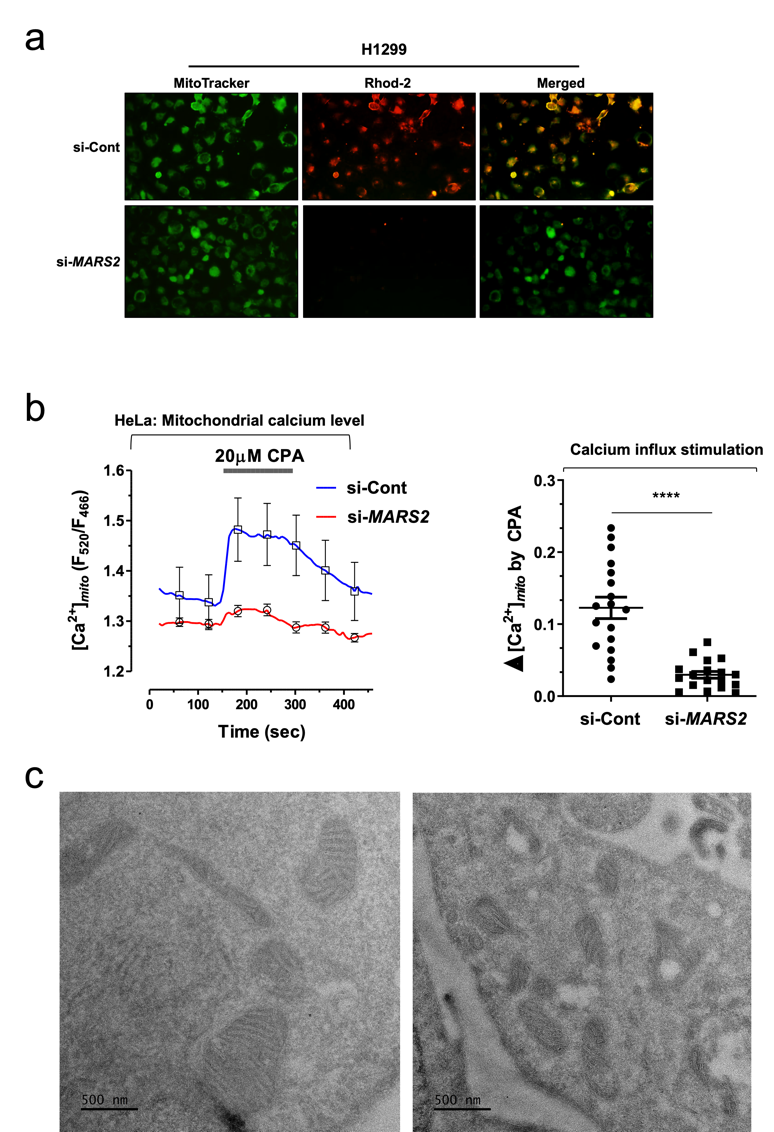
**

**Figure S2. MARS2 regulates mitochondrial Ca^2+^ influx via interaction with MCU. a.** Mitochondrial Ca^2+^ level was visualized by fluorescence microscopy using Rhod-2 upon *MARS2* knockdown in H1299 cells (n = 3). si-Cont indicates si-Control. **b.** Mitochondrial matrix Ca^2+^ level was measured using FRET-based cameleon protein probe 4mitD3 in HeLa cells upon *MARS2* knockdown (left) (n = 18). Stimulation of mitochondrial Ca^2+^ uptake induced by CPA (100 μM) was measured upon *MARS2* knockdown in HeLa cells (right) (n = 18). **c.** Cryo-immunogold microscopy for negative controls (n = 27). For negative controls, we performed the microscopy without primary antibodies. All the quantitative data in graphs are marked as the mean ± S.D from at least three independent samples. Statistical analyses of results were performed with Student’s t-test (****, P<0.0001).

**
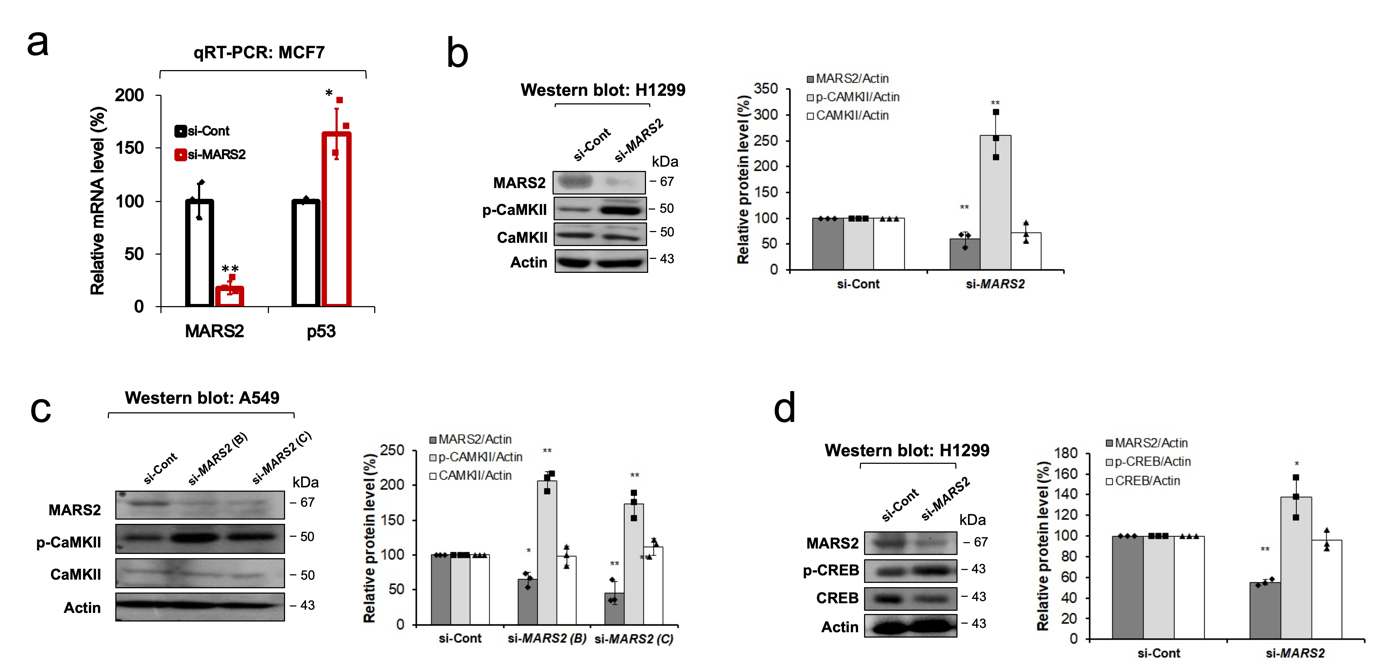
**

**Figure S3. MARS2 regulates p53 via CaMKII/CFREB signaling. a.** Transcriptional expression level was evaluated by qRT-PCR with *MARS2* knockdown in MCF7 human breast cancer cells (n = 9). si-Cont indicates si-Control. **b.** Western blot analysis of CaMKII activation (p-CaMKII: active form of CaMKII) in H1299 cells upon *MARS2* knockdown (n = 3). **c.** Stimulatory effect on CaMKII activation by *MARS2* knockdown was reconfirmed with two different siRNAs. Protein level of p-CaMKII was evaluated by western blot analysis with *MARS2* knockdown using two anti-*MARS2* si-RNAs with different nucleotide sequences (n = 3). **d.** Western blot analysis of CREB activation (p-CREB: active form of CREB) in H1299 cells upon *MARS2* knockdown (n = 3). All the quantitative data in graphs are marked as the mean ± S.D from at least three independent samples. Statistical analyses of results were performed with Student’s t-test (*, P< 0.05, **, P<0.01).

**
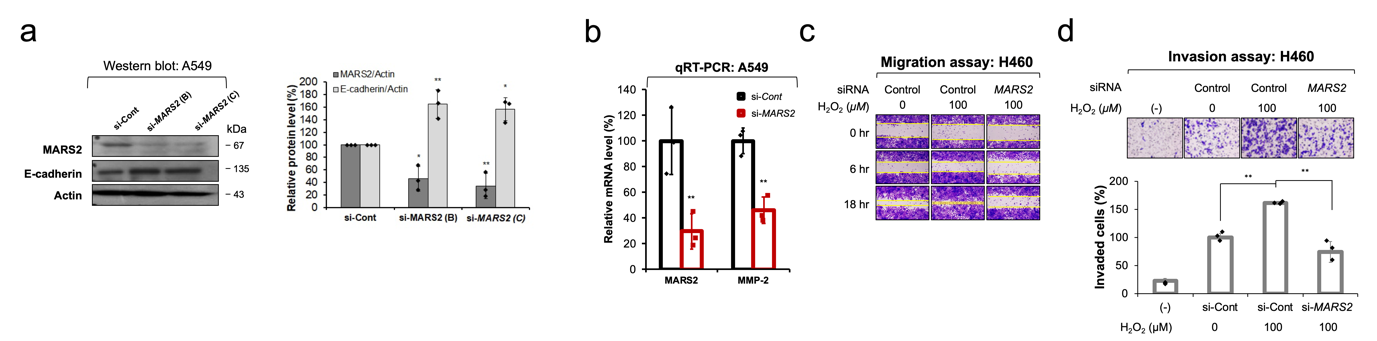
**

**Figure S4. MARS2 regulates EMT.** **a.** Stimulatory effect on E-cadherin level by *MARS2* knockdown was reconfirmed with two different siRNAs. Protein level of E-cadherin was evaluated by western blot analysis with *MARS2* knockdown using two anti-*MARS2* si-RNAs with different nucleotide sequences (n = 3). si-Cont indicates si-Control. **b.** Transcriptional expressions of MMP-2 was evaluated by qRT-PCR upon *MARS2* knockdown in A549 cells (n = 9). **c.** Wound-healing cell migration assay was performed with *MARS2* knockdown and *MARS2* knockdown + H_2_O_2_ (100 μM) in H460 cells (n = 3). **d.** Invasive ability of H460 cells was tested using Boyden chamber assay with *MARS2* knockdown (n = 3). Media containing 0.1% FBS (-) were used as negative controls. All the quantitative data in graphs are marked as the mean ± S.D from at least three independent samples. Statistical analyses of results were performed with Student’s t-test or ANOVA followed by Tukey’s test. (**, P<0.01)

**
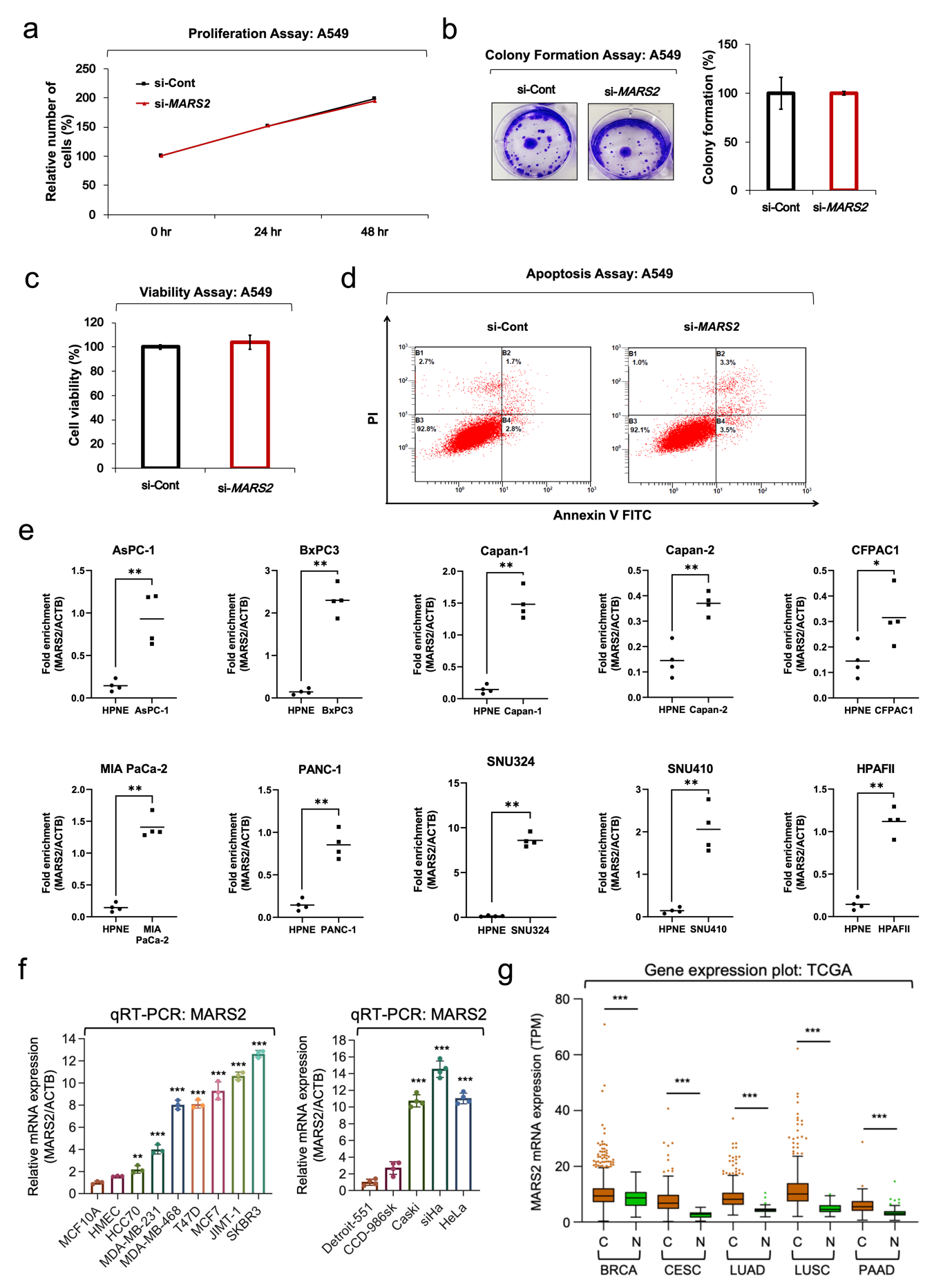
**

**Figure S5. MARS2 is associated with cancer metastasis. a.** Proliferation of A549 cells (n = 3) was not affected by MARS2 knockdown (n = 3). si-Cont indicates si-Control. **b.** Colony formation of A549 cells (n = 3) was not affected by MARS2 knockdown. **c.** Viability of A549 cells (n = 3) was not affected by MARS2 knockdown. **d.** Apoptosis was not affected by MARS2 knockdown (n = 3). **e.** MARS2 is overexpressed in pancreatic cancer cells. Comparisons of MARS2 expression levels in HPNE normal cell *versus* pancreas cancer cell-lines, AsPC-1, BxPC3, Capan-1, Capan-2, CFPAC1, MIA PaCa-2, PANC-1, SNU324, SNU410 and HPAFII (n =4 for each cell line). Statistical analyses of results were performed with Student’s t-test (*, P< 0.05, **, P< 0.01). **f.** The expression profile of MARS2 in normal and cancer cell lines of human breast (left) (normal; MCF10A, HMEC, cancer; HCC70, MDA-MB-231, MDA-MB-468, T47D, MCF7, JIMT-1, SKBR3) and cervix (right) (normal; Detroit-551, CCD-986sk, cancer; Caski, siHa, HeLa) (n = 4). The level of *MARS2* and control *ACTB* mRNA expression was analyzed by qRT-PCR. Data represent the mean values of four independent experiments, and error bars in the graph represent ± standard deviation. For the evaluation of statistical significance, unpaired two-tailed Student’s *t*-test was performed, and the *p*-values compare each cancer cell line to normal cell line (Breast; MCF10, Cervix; Detroit-551) (***p* < 0.01, ****p* < 0.001). **g.** Gene expression analysis comparing MARS2 expression between cancer (brown) and normal (green) samples across various cancers through OncoDB (n = 20). C, cancer; N, normal. Circles outside the boxplot represent outliers. *p*-value was determined by unpaired two-tailed Student’s *t*-test. ****p* < 0.001. BRCA, breast invasive carcinoma; CESC, cervical squamous cell carcinoma and endocervical adenocarcinoma; LUAD, lung adenocarcinoma; LUSC, lung squamous cell carcinoma; PAAD, pancreatic adenocarcinoma.

**Table S1. Sequences of siRNAs used in this study**

| siRNA |  | Sequence (5’ – 3’) |
| --- | --- | --- |
| si-*MARS2* | Sense | GGAUUCGUUUCCUGUAUCUTT |
|  | Antisense | AGAUACAGGAAACGAAUCCTT |
| si-*MARS2* (B) | Sense | GUAGCAGACCACUAUGAUATT |
|  | Antisense | UAUCAUAGUGGUCUGCUATT |
| si-*MARS2* (C) | Sense | CUUACCGGCUUGUGGUAAAT |
|  | Antisense | UUUACCACAAGCCGGUAAGTT |
| si-*TP53* | Sense | CUACUUCCUGAAAACAACGTT |
|  | Antisense | CGUUGUUUUCAGGAAGUAGTT |
| si-*MCU* | Sense | CGACCUAGAGAAAUACAAUTT |
|  | Antisense | AUUGUAUUUCUCUAGGUCGTT |
| si-*ZEB1* | Sense | UGAUCAGCCUCAAUCUGCATT |
|  | Antisense | UGCAGAUUGAGGCUGAUCATT |

**Table S2. List of primary antibodies**

| Antibody | Species | Company |
| --- | --- | --- |
| MARS2 | Mouse | Santa Cruz (sc-293382) |
| COX2 | Rabbit | Abcam (ab79393) |
| COX4 | Mouse | Santa Cruz (sc-376731) |
| p53 | Mouse | Santa Cruz (sc-126) |
| TIGAR | Rabbit | Novus Biologicals (NBP1-49534) |
| p-PDH | Rabbit | Sigma-Aldrich (ABS204) |
| PDH | Mouse | Santa Cruz (sc-377092) |
| MCU | Rabbit | Cell Signaling (#14997) |
| Sym | Mouse | BD Biosciences (Clone 25) |
| Flag | Mouse | BioLegend (Clone L5) |
| H3 | Rabbit | Cell Signaling (#4499) |
| p-CAMKII | Rabbit | Cell Signaling (#12716) |
| CAMKII | Rabbit | Cell Signaling (#3362) |
| p-CREB | Mouse | Santa Cruz (sc-81486) |
| CREB | Mouse | Cell Signaling (#9104) |
| E-cadherin | Mouse | Cell Signaling (#5296) |
| ZEB1 | Rabbit | Cell Signaling (#3396) |
| Actin | Goat | Santa Cruz (sc-1615) |
| Tubulin | Mouse | Santa Cruz (sc-5274) |

**Table S3. Primer sequences used in qRT-PCR**

| Gene |  | Sequence (5’ – 3’) |
| --- | --- | --- |
| *MARS2* | Forward | GCACAGATTTCATCCGCACC |
|  | Reverse | AGGAAACGAATCCCCCGATG |
| *MARS2*  (ChIP) | Forward | GCGATGATGCTTGTGATGTG |
|  | Reverse | AAGATTCAGCAGGCAGCA |
| *GAPDH* | Forward | TGCACCACCAACTGCTTAGC |
|  | Reverse | GGCATGGACTGTGGTCATGAG |
| *MMP2* | Forward | TTGACGGTAAGGACGGACTC |
|  | Reverse | ACTTGCAGTACTCCCCATCG |
| *CDH1* | Forward | ACAGCCCCGCCTTATGATT |
|  | Reverse | TCGGAACCGCTTCCTTCA |
| *SNAI1* | Forward | CCCCAATCGGAAGCCTAACT |
|  | Reverse | GCTGGAAGGTAAACTCTGGATTAGA |
| *SNAI2* | Forward | TTCGGACCCACACATTACCT |
|  | Reverse | GCAGTGAGGGCAAGAAAAAG |
| *TWIST1* | Forward | GGAGTCCGCAGTCTTACG |
|  | Reverse | TCTGGAGGACCTGGTAGAGG |
| *TP53* | Forward | CCCTGTCATCTTCTGTCCCT |
|  | Reverse | GACTTGGCTGTCCCAGAATG |
| *MCU* | Forward | GCAGAATTTGGGAGCTGTTT |
|  | Reverse | GTCAATTCCCCGATCCTCTT |
| *ZEB1* | Forward | GCGATGATGCTTGTGATGTG |
|  | Reverse | TGCTGCCTGCTGAATCTT |
| *ACTB* | Forward | CTGGAACGGTGAAGGTGACA |
|  | Reverse | GGGACTTCCTGTAACA ACGCA |
| *GPx1* | Forward | TGCTCGGCTTCCCGTGCAACCAGT |
|  | Reverse | GGTGATGAGCTTGGGGTCGGTCAT |
